# The Hydration Status of Adult Patients with Oropharyngeal Dysphagia and the Effect of Thickened Fluid Therapy on Fluid Intake and Hydration: Results of Two Parallel Systematic and Scoping Reviews

**DOI:** 10.3390/nu14122497

**Published:** 2022-06-16

**Authors:** Paula Viñas, Mireia Bolivar-Prados, Noemi Tomsen, Alicia Costa, Sergio Marin, Stephanie A. Riera, Núria Barcons, Pere Clavé

**Affiliations:** 1 Gastrointestinal Physiology Laboratory, Hospital de Mataró, Department of Medicine, Universitat Autònoma de Barcelona, 08304 Barcelona, Spain; pvinas@csdm.cat (P.V.); mbolivar@csdm.cat (M.B.-P.); ntomsen@csdm.cat (N.T.); acosta@csdm.cat (A.C.); sergiomarinrubio@gmail.com (S.M.); eriera@csdm.cat (S.A.R.); 2 Centro de Investigación Biomédica en Red de Enfermedades Hepáticas y Digestivas (CIBERehd), 28029 Madrid, Spain; 3 Pharmacy Department, Hospital Universitari Germans Trias i Pujol, 08916 Badalona, Spain; 4 Medical Affairs, Nestlé Health Science, CH-1800 Vevey, Switzerland; nuria.barcons@es.nestle.com

**Keywords:** deglutition, deglutition disorders, dysphagia, hydration, hydropenia, dehydration, nutritional status, aspiration, thickened fluids, thickeners

## Abstract

Background: The effect of oropharyngeal dysphagia (OD) and thickened fluid (TF) therapy on hydration status has not been well defined in the literature. We aimed to assess the hydration status in patients with OD and the effect TF therapy has on it. Methods: Two literature reviews following PRISMA methodology (each one including a systematic and a scoping review) were performed: (R1) hydration status in adult patients with OD; (R2) effect of TF therapy on fluid intake and dehydration. Narrative and descriptive methods summarized both reviews. Quality assessment was assessed by Joanna Briggs Institute tools and GRADE. Results: (R1) Five out of twenty-two studies using analytical parameters or bioimpedance showed poorer hydration status among OD and 19–100% prevalence of dehydration; (R2) two high quality studies (total of 724 participants) showed positive effects of TF on hydration status. Among the articles included, nine out of ten studies that evaluated fluid intake reported a reduced TF intake below basal water requirements. Conclusions: Dehydration is a highly prevalent complication in OD. There is scientific evidence on the positive effect of TF therapy on the hydration status of patients with OD. However, strict monitoring of fluid volume intake is essential due to the low consumption of TF in these patients.

## 1. Introduction

Oropharyngeal dysphagia (OD) is a symptom of the difficulty or inability to form or safely move a bolus from the mouth to the esophagus, and can include aspirations [1]. OD is recognized by the World Health Organization in the International Classification of Diseases (ICD) with code MD93 in the latest version (ICD-11) [2]. OD is a prevalent condition affecting 27% of independent older people and 51% of those institutionalized [3,4]. In 2016, OD was also recognized as a geriatric syndrome by two European societies, the European Society for Swallowing Disorders and the European Geriatric Medicine Society [5].

Dysphagia can cause two main groups of complications: (a) efficacy impairments which include oral and pharyngeal residue, labial seal impairment and inability to form boluses, among others, and can lead to malnutrition and dehydration; and (b) safety impairments, including penetrations and aspirations of the bolus to the respiratory system which can lead to tracheobronchial aspirations and produce respiratory infections and aspiration pneumonia with high mortality rates [6,7,8]. Although dehydration is considered one of the major complications of OD in older people [6], research is scarce and its prevalence in patients with OD is not well described. Some studies have shown the occurrence of hydropenia and reduction in the intracellular water (ICW) compartment and saliva volume in older patients with OD [9,10], which could be the consequence of reduced water intake associated with OD, loss of the sensation of thirst, and loss of body fluids with a lower osmolality with respect to plasma [11]. Patients with chronic OD showed a decrease in ICW compartment [12], but no method has been standardized to evaluate hydration status in these patients. Other authors [13,14] have shown that patients with OD are at high risk of dehydration, which represents a common cause of morbidity and hospital readmissions in this phenotype of patients. Nonetheless, few studies have assessed the hydration status of patients suffering from OD in an objective manner.

Thickened fluids (TF) and the use of thickening products were demonstrated to be a valid therapeutic strategy to reduce the risk of airway invasion in patients with OD [15] in several clinical trials performed by our group [16,17,18,19]. Shear viscosity is the main physical property associated with the therapeutic effect of TF [20]. Increasing shear viscosity of fluids with the use of thickening products increases the prevalence of safe swallows in several phenotypes of patients with OD: older, post-stroke [18,19], head and neck cancer and those with neurodegenerative diseases [19]. In addition, multimodal strategies including TF decreased the rate of respiratory infections and hospital readmissions and improved nutritional status and survival in a 6-month follow-up study [21]. The evidence behind TF preventing aspiration in OD has been recently questioned, and reduced fluid intake associated with fluid thickening has been proposed as a major cause of dehydration in patients with dysphagia [22]. The therapeutic effect of using thickening products to thicken fluids depends on several factors such as the basal hydration status of patients, the composition of TF (thickening agent), the preparation method, the amount of thickening product used, the viscosity obtained and, especially, the thickening product’s behaviour on contact with salivary amylase in the oral phase and with shear thinning caused by bolus velocity in the pharyngeal phase, rheological factors that affect viscosity during swallow [20]. Another factor which should be considered when prescribing thickening products is the low compliance of the patient due to the reduced palatability of these products as viscosity increases [23], and which might reduce fluid volume intake. Finally, the lack of consensus on the definition of and methodology for dehydration diagnosis hinders the study of the effect of OD and TF on fluid intake and dehydration in vulnerable patients.

Our study is the result of two literature reviews, each with a systematic (SR) and a scoping review (ScR), following PRISMA and PRISMA-ScR methodologies, on: (a) the prevalence of dehydration in OD; (b) the relationship between OD severity and dehydration severity; and (c) the effect of TF therapy on hydration status in this population.

## 2. Materials and Methods

**Figure 1:**
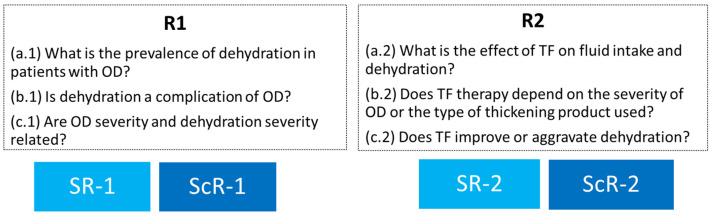
Questions for Review 1 (R1) and Review 2 (R2). SR-1 Systematic Review 1; ScR-1 Scoping Review 1; SR-2 Systematic Review 2; ScR-2 Scoping Review 2.

Two reviews have been designed in this study to answer two groups of PICO (Population, Intervention, Comparison and Outcomes) questions: (R1) the hydration status of adult patients affected by OD; and (R2) the effect of TF therapy on hydration status in this population. More specific questions as described in Figure 1 were developed:

- Review 1 (SR + ScR)—*Hydration status of patients with OD*: a. (1) What is the prevalence of dehydration in adult patients with OD? b. (1) Is dehydration a complication of OD in adult patients with OD? c. (1) Is there a relationship between OD severity and dehydration severity?
- Review 2 (SR + ScR)—*Effect of TF therapy on hydration status*: a. (2) What is the effect of TF therapy on the fluid intake and hydration status of patients with OD? b. (2) Does the effect of TF therapy depend on the severity of OD or the type of thickening product used? c. (2) In patients with OD, does treatment with TF improve or aggravate dehydration?

Both reviews include an SR and an ScR to explore the literature. The protocols of both reviews have been previously registered (PROSPERO) (https://www.crd.york.ac.uk/prospero/, accessed on 1 October 2020) under codes: CRD42021272030 (R1) and CRD42021242098 (R2). SRs were carried out using the Preferred Reporting Items for Systematic Reviews and Meta-Analyses (PRISMA) [24] methodology and ScRs, PRISMA extension (PRISMA-ScR) [25]. Task organization in these reviews including those processes performed by two or more authors (selection of studies, data extraction, and quality assessment) are explained elsewhere (Supplementary File S1).

### 2.1. Patient and Public Involvement Statement

There was no public or patient involvement in the elaboration of these reviews.

### 2.2. Search Strategy

We searched MEDLINE using PubMed, Embase using Ovid and Web of Science until 31 May 2021. The combined Mesh and search terms used in PubMed searches are described in Supplementary Table S1 (R1) and Supplementary Table S2 (R2) from the protocol (Supplementary File S1). Equivalent search strategies were applied to subsequent searches. The reference lists of the selected articles were checked to find additional eligible studies. No publication date or language restrictions were imposed. Unpublished material, pre-prints, posters, protocols, surveys, book chapters, doctoral theses and abstracts were not included.

A double-phase process was used, involving a first screening phase and a subsequent final selection phase according to the eligibility criteria. In the first screening phase, the abstract and title of studies were analyzed to eliminate those that did not have minimally relevant information on the hydration status of adult patients with OD (R1) or on the effect of TF treatment on hydration status and fluid intake in these patients (R2). This selection process was performed independently by two reviewers and, in the case of disagreement between them, the opinion of a third reviewer was sought in order to reach a consensus and make a final decision.

In the second selection phase, the full text of the selected articles was evaluated and included according to the eligibility criteria.

### 2.3. Eligibility Criteria and Selection Process

Review 1—*Hydration status of patients with OD*: We included cross-sectional, cohort, case-control (studies in which the cases were patients with OD and the controls were patients without OD) and randomized studies if they reported information on adult patients (≥18 years) affected by OD (assessed by clinical and/or instrumental method) in which hydration status was assessed using an objective method (such as analytical measurements or bioelectrical impedance analysis (BIA)) for the SR. Articles were excluded if patient hydration status was not assessed or if they referred to esophageal dysphagia. These and review articles were included in the ScR, as well as cross-sectional, cohort, case-control and randomized studies in which hydration status was assessed using subjective methods. The main outcome of interest was the hydration status in adult patients with OD.

Review 2—*Effect of TF therapy on hydration status*: We carried out a second SR and ScR to identify the effect of TF therapy on fluid intake and hydration status of patients with OD. The SR included cross-sectional, cohort, case-control (studies in which the cases were patients with OD and the controls were patients without OD) and randomized studies in which hydration status was assessed using an objective method (such as analytical measurements or bioelectrical impedance analysis) and the effect of TF therapy was reported in adult patients (≥18 years) affected by OD (assessed by clinical and/or instrumental method). The ScR also included review articles, as well as cross-sectional, cohort, case-control and randomized studies in which the effect of TF therapy on hydration status or fluid intake was assessed using subjective methods. 

### 2.4. Data Presentation and Summary Measures

Data were reported in their original format using tables and narrative. A narrative method was used to synthesize this evidence (Supplementary File S1).

### 2.5. Quality Evaluation and Strength of the Evidence

For quality assessment and risk of bias, we used the critical appraisal tools provided by the Joanna Briggs Institute (JBI) [26] specific for each type of study included. JBI checklists provide a set of items to assess the trustworthiness, relevance and results of studies to be included in an SR. For each study, the total number of items was rated as “yes (1 point)”, “unclear (0.5 points)” and “no (0 points)” and then divided by the total number of items applicable. This total score is presented as a percentage in which a higher score represents a lower risk of bias. Rates were defined as: insufficient [0–33%], sufficient (33–66%] and high (66–100%] quality. Quality assessments of the studies in both reviews are presented as Supplementary Material (Supplementary File S2). In addition, we rated the strength of evidence across studies as high, moderate, low or very low using Grading of Recommendations Assessment, Development and Evaluation methodology (GRADE) [27]. As explained in the protocol (Supplementary File S1), quality assessment, risk of bias and strength of evidence were performed in both SR, but are not required in an ScR.

## 3. Results

**Figure 2:**
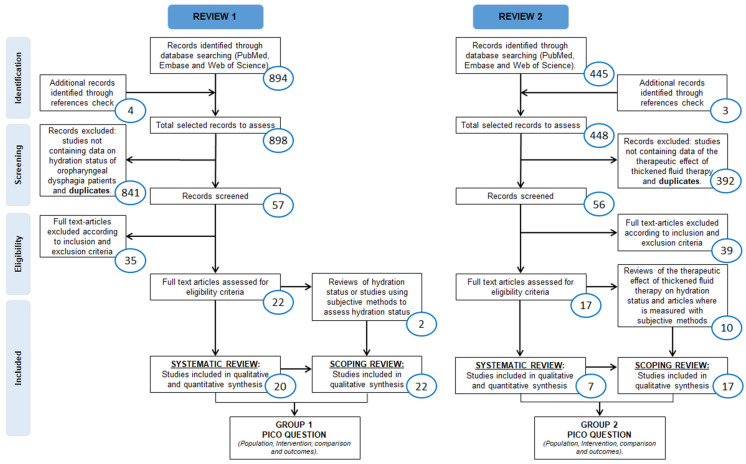
R1 and R2 flowcharts following the PRISMA guidelines including a systematic review and a scoping review for each one.

Figure 2 shows the two parallel flow charts describing the selection processes and inclusion criteria following PRISMA guidelines in each review. A total of 22 articles were included in Review 1 (SR *n* = 20; ScR *n* = 22), and 17 articles in Review 2 (SR *n* = 7; ScR *n* = 17). The endpoints of the flowchart correspond to the answers to the questions selected for each review.

### 3.1. Review 1—Hydration Status of Patients with Oropharyngeal Dysphagia

A total of 894 articles were identified using the search terms (649 through MEDLINE using PubMed, 226 through Embase using Ovid and 19 through Web of Science) and 4 articles were identified through reference checks. After screening both title and abstract of these articles, 841 were excluded because they did not provide minimal relevant information on the assessment or status of hydration in patients with OD: 43 articles were assessed in animals, 250 did not assess OD, 344 did not evaluate hydration status, 44 did not recruit patients suffering from OD and also the hydration status was not evaluated, and 19 studies did not relate or link OD and dehydration; 137 articles were duplicates. A second evaluation phase following eligibility criteria was carried out with the 57 remaining studies and 35 of them were excluded. Of the 22 remaining articles, 20 were included in the SR and 22 in the ScR.

#### 3.1.1. Systematic Review (SR-1)

Twenty studies assessed the hydration status in adult patients with OD using an objective method. Of these, 11 studies were conducted exclusively in stroke patients, 6 studies in older patients and 3 included several OD phenotypes (stroke and other neurological pathologies, cancer, autoimmune and infectious diseases). Of these 20 articles, only 5 [9,10,28,29,30] used bioelectrical impedance analysis to assess hydration status; 4 of them measured body water compartments to assess hydration status (total body water (TBW), ICW, extracellular water (ECW) and/or the relationship between ECW and ICW (ECW/ICW); the analysis of phase angle (PA°) was also used in 2 studies, and the remaining 15 articles [13,14,31,32,33,34,35,36,37,38,39,40,41,42,43] used biochemical data as an objective method to assess hydration status. Characteristics of each study are reported in Table 1: OD etiology and instrumental assessment, hydration parameter used, number of participants and phenotype, main conclusions of the study and quality assessment. Due to the heterogeneity of the results, a meta-analysis was not performed.

*Bioimpedance studies*. A cross-sectional study by Carrión S et al. [9] showed that older patients with chronic OD presented a significant decrease in ICW compared with older healthy persons without OD. A second cross-sectional study from the same group [10] compared two groups of healthy persons (G1: 18–55 years and G2: >65 years) vs. a group of older patients with OD (G3) showing a significant reduction in the ICW (%) and in the PA° when comparing G3 with G1. Data provided by the corresponding author showed that 67% of older people with OD presented values of PA° suggesting dehydration compared with 50% of older patients without OD. A study by Goldberg A et al. [28] on older women in residential care (six of them suffering from OD) reported that all the women presented reduced TBW compared to the expected values for 80–90-year-old women published by Chumlea et al. (2002) [44]. A randomized trial performed by Sezgin B et al. [29] on patients submitted to total maxillectomy found that pre-intervention prevalence of OD, ranging between 40% and 60%, increased after the surgical procedure to 70–80%. Hydration status was also assessed for the whole group measuring ICW, ECW and TBW pre- and post-intervention, and a significant worsening in hydration status was observed between values. A cross-sectional study by Ramos-Vázquez et al. [30] on 54 patients with OD needing fluid adaptation and 25 patients with OD and exclusive tube feeding found that this last group of patients had significantly lower PA° (3.7 ± 0.9) compared to the patients on nectar and spoon-thick viscosities (4.6 ± 1.1; *p* = 0.005), concluding that most severe OD patients presented lower PA° and poorer hydration status.

*Biochemical studies*. Blood urea nitrogen to creatinine (BUN/Cr) ratio was the most common analytical parameter used to assess hydration status [13,14,32,33,34,35,36], followed by BUN [14,34,36,37,38] and serum sodium concentration [14,32,34,36,37,39]. Other analytical parameters used were urea, hematocrit, urine/serum osmolality, urine sodium and serum creatinine [14,32,39,40,41]. One of the articles included in this review did not specify the biochemical parameter used for the analysis [42]. The main characteristics and quality assessment of the studies are described in Table 1.

Among the studies that found a significant association between OD and dehydration, Lee A et al. [42] studied 211 hospitalized older patients, of whom 62 had swallowing impairments, and found that these latter patients had a significantly increased risk of dehydration (RR 2.82). Churchill M et al. [36] assessed the effects of the use of diuretics on hydration status in 296 post-stroke patients (20.61% with OD) and concluded that having OD and/or having thin-liquid restriction were associated with significantly higher peak BUN and sodium levels. In 2013, Crary M et al. [31] showed that hospitalized stroke patients with OD (*n* = 25) had significantly higher BUN/Cr ratio values compared with patients without OD (*n* = 42). The same author [13] studied 67 acute stroke patients with 38% prevalence of OD. OD patients presented significantly higher mean BUN/Cr values than those without OD according to BUN/Cr ratio > 20:1. For studies including only OD patients, Leibovitz et al. [14] found that 75% of 28 patients with Grade 2 feeding difficulties presented significantly more dehydration parameters compared with 67 nasogastric-tube-fed patients. In 2018, Howard M et al. [38] made a retrospective evaluation of 20 OD patients admitted to a rehabilitation facility. On admission, their BUN, serum creatinine and sodium levels were above the acceptable levels, pointing to signs of dehydration. Murray et al. [35] studied 14 OD patients from a stroke unit, and 71% of them presented a BUN/Cr ratio > 20, suggesting dehydration.

In contrast, other studies failed to find any significant association between OD and dehydration. Schmidt J et al. [37] compared the hydration status of two groups of post-stroke patients according to whether there was an aspiration during videofluoroscopy (VFS). Of 59 patients, 26 presented aspiration, and 19.20% of them were dehydrated, compared with 9.10% in non-aspirators (*p* = 0.20). Smithard DG et al. [41] examined the hydration status of 121 patients after an acute stroke on days 0 to 7. Up to 50% of the patients assessed presented OD, but no changes in the hydration status were observed. A cross-sectional study from Botigué et al. [32] studied the deglutition and hydration status of 53 institutionalized older patients and found that 83.3% of patients with OD had BUN/Cr > 21 compared with 74.29% in non-OD patients, without significant differences between both groups. Buoite SA et al. (2019) [39] described the hydration status of stroke patients during acute hospitalization in a stroke unit, and no significant differences in hydration status between patients with (*n* = 18) and without OD (*n* = 56) were observed, neither on admission nor discharge. A study from Murray J et al. [33] investigated the hydration status of 100 patients in stroke rehabilitation facilities by determining the BUN/Cr ratio in two groups: 14 OD patients vs. 86 patients without OD. No significant differences were observed, and OD was not considered a significant predictor of the hydration outcome. Goroff H et al. [34] developed a retrospective observational study on 712 patients with an initial or recurrent ischemic stroke which assessed the hydration status during admission to an acute care hospital for patients with (using TF) and without OD. No significant differences were observed in serum sodium for any group of patients from the admission and discharge except for the BUN and BUN/Cr ratios which were mildly increased on discharge. In 1998, Sala R et al. [40] also assessed OD prevalence and hydration status on admission (OD: 36%) and discharge (OD: 12.83%) in a sample of 187 patients with cerebrovascular accidents. Prolonged admissions and OD led to a lower increase in urea, which was explained by the obligatory use of perfusions or nasogastric tubes in more severe patients. A more recent study from Kim et al. [43] included 52 non-neurologically ill patients who underwent VFS in rehabilitation facilities. Up to 50% of them presented airway invasion during the exploration. No significant difference in the BUN/Cr ratio was observed in comparison with patients without airway invasion.

#### 3.1.2. Scoping Review (ScR-1)

Two additional studies were included in the ScR. The first, a publication from Via M et al. (2013) [45], stated that patients with OD are at high risk of dehydration but prevalence is unknown due to: (a) difficulty in quantifying hydration status; and (b) there is no agreement over a standard clinical definition. They suggested that an elevated BUN/Cr ratio (>15) is useful for the diagnosis of dehydration. A more recent SR from Schettino et al. (2019) [46] reviewed the hydration status in post-stroke patients in 18 different studies, concluding that patients with OD had an increased BUN/Cr ratio and decreased water intake, indicating worsening hydration status. Authors reported that prevalence of dehydration in post-stroke patients during hospitalization can range from 11% to 66%, and suggested that in the initial phase of stroke, the change in hydration status may be a consequence of a decreased water intake due to presence of OD. The rest of the studies included in ScR-1 have been commented above [13,31,33,34,35,36,47].

#### 3.1.3. Synthesis of the Studies’ Findings on the Hydration Status of Patients with OD

*Hydration status of patients with and without OD*: Five studies showed a statistically significant relationship suggesting that patients with OD are more dehydrated than the ones without—one using BIA and four using biochemical parameters [9,13,31,36,42]. Some other studies reported high prevalence of dehydration in groups of older patients with and without OD with no significant differences between them [10,28,32,34,35,37,39,40,43].

*Relationship between OD severity and hydration status*: We found only two studies [14,30] comparing the hydration status in patients affected by OD of varying severities. Their results found significantly poorer hydration status in patients with more severe OD.

*Prevalence of dehydration in patients with OD*: Prevalence of dehydration in patients affected by OD was found in 19–100% of patients [9,10,14,28,32,35,37,42].

#### 3.1.4. Quality and Strength of Evidence across Studies

The quality of all studies assessing the hydration status of adult patients with OD in the SR were considered as at least sufficient with the JBI. Total scores for each study are available in Table 1, and specific results for each study are available in the online Supplementary Appendix (Supplementary File S2). Of the studies comparing hydration status of patients affected by OD with the ones without, nine prospective and six retrospective studies were assessed. Quality of the studies was at least sufficient for all of them. High consistency was found among the results of thirteen studies and a direct effect was found in all except for two of them. Regarding prevalence of dehydration, nine studies were assessed (eight prospective and one retrospective). The risk of bias was low in seven studies and moderate in two. Consistency in the results was found in all except one study and the same for direct effects. Finally, two studies with a low risk of bias and high consistency evaluated the relationship between severity of OD and dehydration severity.

### 3.2. Review 2—Effect of Thickened Fluid Therapy on Hydration Status

A total of 445 articles were initially selected from the database search (PubMed: 205 articles; Embase: 32 articles and Web of Science: 208 articles) and 3 additional studies were identified by checking bibliographic references, giving a total of 448 articles in which title and abstract were screened. Only 17 passed the eligibility criteria: 7 of them were included in the SR-2, and 17 in the ScR-2. Figure 2 shows the flow chart of the selection process.

#### 3.2.1. Systematic Review (SR-2)

Six studies evaluated the hydration status by biochemical methods in patients affected by stroke [13,34,35,38,47,48]. One study was carried out on patients with a maxillary carcinoma and used BIA to assess the hydration status [29]. All the studies were conducted on patients using some TF therapy explained below. The main characteristics are described in Table 2. Due to the heterogeneity of the results, a meta-analysis was not performed.

*Bioimpedance study*. Concerning the use of BIA to assess the hydration status of OD patients, the randomized clinical trials of Sezgin B et al. [29] evaluating the role of a xanthan-gum-based thickener on hydration status in 12 patients who underwent a total maxillectomy were also included in this second review. They concluded that using a xanthan-gum-based thickener helped maintain ICW, ECW and TBW after 3 months post-total maxillectomy compared with the control group (Intervention group: 24.60 ± 4.70, 18.45 ± 2.86 and 43.05 ± 7.05 vs. Control group: 20.55 ± 3.73; 16.38 ± 1.03 and 36.93 ± 4.56, respectively; *p* < 0.05 each).

*Biochemical studies*. Goroff et al. [34] carried out a retrospective observational study with 712 post-stroke patients during their rehabilitation hospital stay. During this period, they received specific strategies to increase their hydration and modify liquid consistency with thickening products. On discharge, their hydration levels (serum sodium, BUN and BUN/Cr ratio) were stable or slightly improved for all liquid consistency modification treatments. A study from DePippo KL et al. [48] followed 115 patients with OD after a stroke and randomized them into three interventions (G1: all liquid consistencies; G2: TF; G3: daily evaluation during meals). The prevalence of dehydration was higher in G1; however, no significant differences were observed between groups (G1: 7.9%, G2: 0%, and G3: 2.6%). Whelan K et al. [47] evaluated the hydration status in 24 patients with OD in the acute phase of stroke who were randomly assigned to receive thickening products (control group = 13) or pre-thickened drinks (intervention group = 11). Results showed 8.3% of hypernatremia, 50% of hyperuricemia, and 12.5% of hypercreatininemia in all the samples. Authors also monitored urinary tract infections, finding no statistical differences between groups. Murray J et al. [35] studied 14 post-stroke patients with OD who were randomized into two groups (G1: only access to TF, and G2: access to TF and thin liquids). The hydration status according to BUN/Cr ratio in G1 decreased (Day 0: 20.28 ± 3.88; Day 14: 24.70 ± 12.71) compared with G2, which demonstrated a trend to improve in 2 weeks follow-up (Day 0: 22.46 ± 3.70; Day 14: 20.56 ± 3.70). No significant differences were observed between groups, except for the prevalence of urinary tract infections which was significantly higher in G1. Crary M et al. [13] made a retrospective chart review of 64 acute stroke patients [32], finding that the hydration status of patients with OD worsened from admission to discharge (BUN/Cr: 20.54 to 26.32). Patients in both subgroups received TF (75% OD vs. 30% non-OD). Authors concluded that viscosity and textural modification were closely associated with poor hydration status. Howard M et al. [38] analyzed the hydration status of 20 patients with OD admitted to a rehabilitation facility. Results showed that the increment in viscosity of fluids was related to a higher dehydration status. Moreover, a significant decrease in BUN values was observed when viscosity was decreased.

#### 3.2.2. Scoping Review (ScR-2)

A total of 17 articles were included in the ScR-2. Seven of them were also included in the SR-2 and explained above [13,29,34,35,38,47,48], and three of them also assessed fluid intake [35,38,47]. Seven additional articles aimed to evaluate the effect of oral fluid intake on the hydration status of patients with OD [49,50,51,52,53,54,55]. Main characteristics and conclusions are described in Supplementary Material (Supplementary Table S3). Finally, three review articles were included [56,57,58].

Garon B et al. (1997) [49] compared the thirst sensation of two groups of patients with OD (G1: only allowed TF, and G2: TF and free access to water). Up to 90% of participants were displeased with TF. The same intervention groups were used in the study published by Karagiannis M et al. [50], where significant differences were observed in the mean fluid intake with higher values for G2. This group also reported better results according to the satisfaction level, thirst sensation and mouth cleanliness. Results of the study published by McGrail A et al. in 2012 [51] and in 2014 [52] are also in line with these results. In 2014, Karagiannis performed another study [53] pre- (only access to TF) and post-intervention (TF, free access to water and oral hygiene protocol) for a group of patients. Differences also appeared between both interventions. In contrast, Howard M et al. [38] described the satisfaction of a group of patients when consuming textured thin liquids who reported a significant improvement related to thirst sensation. Whelan K et al. [47] reported insufficient fluid intake in a group of patients receiving TF and inability to achieve their daily fluid requirements (22%). The use of supplementary enteral or parenteral fluids did not accomplish the requirements either (59%). McCormick et al. [54] compared differences in fluid intake between the use of pre-TF and thickening powder, finding higher values of fluid intake in patients prescribed with pre-TF. A different design was proposed by Vivanti A et al. [55], who evaluated mean fluid intake by administering daily fluids only by thickened beverages or by thickened beverages and food. Results showed that individuals receiving fluid intake only did not reach the minimum calculated fluid requirements. Murray et al. [35] also presented a comparison composed by a group with free access to water and another with only TF. However, in contrast to the results presented above, the total beverage intake of participants with free access to water was not higher than for those who consumed TF only. In addition, overall satisfaction was similar between groups. In addition to the articles mentioned above, three reviews were included.

An SR from Beck AM [56] aimed to report an update on the effect of TF on several outcomes, including dehydration. They concluded that an increment in the viscosity of liquids did not increase the risk of dehydration; however, patients still preferred thin liquids. Painter V [57] reviewed the evidence for textured modified foods and TF in patients with dementia. Prevalence of dehydration increased with the increment in fluid viscosity ranging from 2.3% to 5.9% in patients prescribed with thin liquids and in patients prescribed with thickened viscosities, respectively. Nevertheless, the authors pointed out the need to further explore the relationship between textured modified foods and other outcomes such as malnutrition or morbidity. Finally, in 2013, Cichero et al. [58] reviewed the literature on the impact of TF on hydration and other outcomes. The main focus of this review relied on the study from Leibovitz et al. (2007) [14], which is also included in our SR-1 and explained above. Cichero et al. also reviewed the studies from Garon (1997) [49] and Vivanti (2009) [55], which report that fluid consumption is reduced in individuals using TF [58]. Authors suggested that the combination of low palatability and poor thirst-quenching ability may be the reason for the low TF consumption, reporting a lower fluid intake with the increment of viscosity [58].

#### 3.2.3. Synthesis of the Studies’ Findings

Of the studies selected for this SR-2, two studies [29,34] described a positive effect of TF—one using xanthan gum and the other modified starch and maltodextrin—on hydration status of dysphagic patients, including a total sample of 724 patients for both studies and a quality of studies over 90%. Three of them [35,48] included 153 patients with a quality over 80% and have been considered neutral as results are inconclusive. The two remaining studies [13,38] described negative effects of TF (using starch-based thickeners) on hydration status in OD. Nonetheless, there were 84 patients included in those negative studies and quality of studies ranged from 60–65%. We also found nine studies describing insufficient fluid intake in patients receiving TF [47,49,50,51,52,53,54,55]. Two studies reported the use of enteral and parenteral support because their patients did not achieve their normal hydration requirements with oral fluid intake only [47,55]. One study did not observe lower fluid intake from TF compared to normal fluids [35]. Pre-TF was more accepted in comparison with thickening powder [54]. Table 3 describes the quality of each study according to the JBI checklist, with the number of participants assessed and how they respond to the PICO questions for SR2.

#### 3.2.4. Quality and Strength of Evidence across Studies

Quality of all studies assessing the effect of TF therapy on the hydration status in patients with OD in the SR were considered at least as sufficient with the JBI. Four prospective and three retrospective studies were assessed in order to examine the effect of TF on hydration status. High quality was observed in the studies, suggesting positive and neutral effects of TF. Two studies with a moderate risk of bias reported negative effects of TF on hydration status. High consistency in the results was only observed in four studies.

## 4. Discussion

The main conclusion of this study is that dehydration is a highly prevalent complication in several phenotypes of patients with OD. Results from SR-1 showed that prevalence of dehydration in OD assessed by objective BIA or biochemical methods ranged from 19–100%. Although the exact prevalence of dehydration in OD is not clear, most studies suggested OD patients were at higher risk for this complication. However, studies also showed that prevalence of dehydration was also very high in older non-dysphagic patients. Therefore, some of the studies have described no significant differences between OD and non-OD patients with respect to these high rates of poor hydration status. Studies included in the ScR also highlighted the need to standardize the biochemical and/or BIA markers to assess and monitor the hydration status of patients with dysphagia. In SR-2, scientific evidence on the positive effect of TF therapy on the hydration status of patients with OD was also found, with high quality studies including a large number of patients with dysphagia. Most studies reported low consumption of TF in patients with OD, so strict monitoring of fluid volume intake is essential to improve hydration status of patients with OD.

OD has been recognized as a geriatric syndrome due to its high prevalence and its relationship with many comorbidities and their poor outcomes, including malnutrition, respiratory infections and aspiration pneumonia, functional disability and frailty, institutionalization and increased readmissions, and mortality [59]. Among older patients with OD, the prevalence of malnutrition can go from 16% in patients with neurogenic dysphagia to 45.3% in hospitalized patients in an acute geriatric unit [59]. The annual incidence of lower respiratory tract infections in patients with signs of impaired safety is significantly higher in comparison with persons without these signs (40.0% vs. 21.8%; *p* = 0.030; OR = 2.39) [7]. OD has also been found to be an independent risk factor for community-acquired pneumonia in older patients, and impaired safety of swallow, a prognostic factor of mortality in these patients [60]. Finally, although dehydration is a well-recognized complication of OD [1], very few studies addressed the topic of diagnosis, treatment and complications of dehydration in older patients with dysphagia, and dehydration has been considered an adverse event of fluid thickening and texture modified diets in patients with dysphagia [22].

Impaired alertness, dietary and fluid intake restrictions, as well as the inability to safely and effectively ingest fluids and/or food, are well known risk factors for poor hydration status [61]. The extent to which age-related changes in total body water, thirst perception and renal concentrating ability increase the vulnerability to dehydration in older patients relative to younger individuals should not be underestimated [62]. An adequate supply of water and fluids are essential to maintain cellular homeostasis and several physiological functions [63]. Dehydration is a loss of water resulting in a body water deficit relative to sodium [64]. The increase in sodium causes plasma osmolality to increase, reducing intracellular volume. This is often referred to as hypovolemic hypernatraemia or hypertonic dehydration. Hypotonic and isotonic dehydration differ from a pathophysiologic point of view, characterizing volume depletion (loss of sodium from the extracellular volume) rather than dehydration. Dehydration is considered to be one of the ten most frequent diagnoses for hospitalization in older adults (≥80 years) and the second most common comorbidity, occurring in 14% of all hospitalizations [65]. Since 1994, Warren et al. reported dehydration in 6.7% of hospitalized patients aged 65 years and over [66]. The range of dehydration prevalence has been shown to be from 60% in community-dwelling adults [67] and to 48% in older adults visiting emergency departments [68]. Our results reported that the prevalence can range from 19–100% in patients with OD, according to analytical parameters and BIA. Reber E et al. reported similar numbers [12]. The true prevalence of dehydration is difficult to quantify because it depends on the indicators used to define it and, as there is no gold standard, it is based on clinical observations in combination with laboratory findings, the most common being the BUN/Cr ratio [69]. Hypertonic/hypernatremic dehydration is the most severe and common type in older people, as in patients with OD, it can increase mortality by up to 42%. It is characterized by hypernatraemia (Na^+^ > 145 mEq/L), hyperosmolarity (plasma osmolarity > 295 mosmol/kg) and a BUN/Cr ratio ≥ 20 [70]. BIA has been suggested as an alternative to the subjective and biochemical indicators of hydration status [71]. In our study, we found a strong relationship between OD and dehydration in several phenotypes, as hydration status was found to be significantly poorer in older patients with OD than without OD, further supporting the high prevalence and potential clinical relevance of dehydration as a complication of OD in older patients.

We also found a wide divergence in how hydration is assessed and diagnosed in patients with dysphagia. Some authors suggest that a BUN/Cr ratio > 15 is sufficient for diagnosis, while others state that a combination of clinical history, clinical observations, laboratory tests and physical assessment is necessary [72]. Parameters from BIA, such as ICW and PA°, should also be considered in the diagnosis of dehydration [73,74]. The results of our review are mostly based on analytical parameters, the BUN/Cr ratio being the most commonly used. However, we also found some studies that used BIA in the diagnosis of dehydration. None of our studies used a combination of both tools.

There is also little information on the relationship between the severity of OD and the prevalence and severity of dehydration. Dehydration may worsen complications in patients with OD, including poor muscle function, lethargy, mental confusion, and further increase the risk of aspiration [6]. Our results also suggest that patients with more severe OD present poorer hydration status [14,30]. However, many of the studies reported that patients with more severe OD are also managed with non-oral methods to preserve hydration status, so the results may be biased [40,41,47].

Treatment of dehydration in patients with OD is also a matter of controversy. Studies support the use of TF mainly as a valid therapeutic strategy to reduce the risk of airway invasion caused by OD [15,16,17,18,19,75,76,77]. However, it has been hypothesized that TF provided to reduce aspirations in patients with OD might also exacerbate their dehydration status [22]. An SR from Beck AM [56], also included in our ScR-2, concluded that it is “good clinical practice” to offer textured modified foods to older people with signs of OD and/or chewing difficulties as a compensatory strategy to support adequate dietary intake. A study from our group using a multimodal intervention, including fluid thickening with MS and texture modified foods, clearly showed a positive effect on clinical outcomes of older patients with OD, reducing the incidence of respiratory infections and improving nutritional status, quality of life and morbimortality, although hydration was not specifically assessed [21]. Two of the studies included in SR-2 also described a positive effect of TF therapy on the hydration status of patients with dysphagia, one using xanthan gum and the other modified starch and maltodextrin [29,34]. One of the studies used specific strategies to increase the volume of fluids offered with significantly positive results in terms of hydration status [34], suggesting that an optimal hydration protocol is necessary for good control of hydration status. Of particular relevance, only a few studies included in our SR-2 monitored the adequacy of the volume of TF intake. One of the studies with the strongest positive effect of TF on dehydration [34] used up to three active strategies to increase fluid intake. In addition, nine of the studies included in ScR-2 monitored the volume of TF intake in patients with OD, noting that TF intake alone does not meet basal hydration requirements without the use of enteral and parenteral fluids [47,55]. In cases where patients are given the option of free access to water, they tend to increase the amount of fluid intake [49,50,53] and drink greater amounts when given thin liquids rather than TF [51,52]. Only one study [35] observed no difference in fluid intake when given the option of free water consumption. It is also noteworthy that patients consume greater amounts when receiving pre-TF compared to regular powder thickening products [54]. Some studies reported the lack of training of nurses and insufficient support during mealtimes, leading to reduced patient access to TF [14,78,79,80], which has also been reported in the ScR-2 [51]. All these results show fluid intake in patients with OD is significantly reduced due to several different factors, and not only because of the intrinsic effect of TF on swallow physiology, but more importantly due to the way TF are prescribed, used and monitored. Regarding our two last PICO questions in R2, we could not find any data linking the effect of TF therapy with the severity of OD or the type of thickening product used, and we found few studies where intensive treatment with TF improved dehydration in patients with dysphagia.

Our review also shows the need for prospective studies to provide clear evidence on the effect of TF therapy on the hydration status of patients with OD. The optimal design of these studies should include an experimental design of two-arm randomized clinical trials: (1) an intervention group with xanthan-gum-based thickener to an optimum viscosity level [18] and with recommendations on how to prepare TF, strategies to improve consumption of these fluids, ensure fluid requirements, and with a 1-month hydration plan; (2) a control group with xanthan-gum-based thickener without strict recommendations nor a hydration plan. The study would include a baseline and 1-month post-intervention assessment of hydration status by BIA and analytical parameters. Strict monitoring of fluid intake and compliance with TF prescription should be established in order to assess the acceptability and palatability of TF. Hydration status should include minimally invasive tools. A consensual guideline is needed to diagnose and treat the hydration status of patients suffering from OD in an objective manner. The combination of ICW and PA° analysis with BIA and BUN/Cr ratio as an analytical parameter can become a good future strategy for the diagnosis of dehydration, not forgetting to establish valid clinical endpoints such as nutritional and hydration status, respiratory complications, palatability, mortality and quality of life.

Study limitations. There are some limitations to our research. The main one is that several studies included and compared patients of different aetiologies or with different interventions. It was not possible to perform a meta-analysis. In addition, some authors suggested that the lack of conclusive results could be due to the low number of participants. Moreover, there were several ways of assessing hydration status, both with analytical parameters and with BIA, and each subgroup used different parameters, so there was no standard diagnostic method. It should be noted that in some studies with patients with varying severities of OD, we were not able to observe changes in their hydration status, probably because the more severe patients were receiving supplementary enteral or parenteral fluids.

## 5. Conclusions

With this study, we aimed to find the prevalence of dehydration in OD, the relationship between OD severity and dehydration severity, the effect of TF therapy on hydration status in OD patients, and any potential negative or positive effect of TF therapy on hydration status. Dehydration is a highly prevalent complication observed in 19 to 100% of patients with OD, and the best biomarkers to monitor this condition are BIA and/or biochemical methods, including ICW, PA° and BUN/Cr. We also found that patients with a higher severity of OD have a worse hydration status; however, further studies are needed to confirm this. We have high quality studies that support the favorable effect of TF in maintaining hydration status in these patients; however, we have observed that TF intake is insufficient, and basal water requirements are not met in many studies. Strict monitoring and support of fluid intake is recommended.

## Figures and Tables

**Table 1:** Main results on hydration status in Systematic Review 1.

Study	OD Etiology	Assessment for OD	Hydration Assessment Parameter	Number of Participants	Conclusion	QA (%) ^1^
Bioimpedance Studies						
Carrión S. [9]	Geriatric, neurologic, other.	VFS, V-VST	ICW, ECW, TBW	133 (A = 95 older with OD (chronic NRL or aging) + B = 23 older with OD (CAP) + C = 15 healthy older people)	Older patients with OD presented a significant reduction in ICW compared to healthy older people.	75.00
Tomsen N. [10]	Geriatric	VFS	ECW, ICW, TBW, ECW/ICW, PA°	43 (A = 15 young healthy + B = 14 healthy older + C = 14 older with OD)	Both older groups showed a significant reduction in ICW, ECW, ECW/ICW ratio and PA° compared to young healthy people.	81.25
Goldberg LR. [28]	Stroke	SLP clinical assessment and FOIS	TBW (kg)	19	The mean levels of TBW for both groups were lower, indicating risk for inadequate hydration in OD.	80.00
Sezgin B. [29]	Maxillary carcinoma	EAT-10, MDADI, FOSS, FOIS	ECW, ICW, TBW	10	After total maxillectomy, the prevalence of OD increased and hydration worsened (significant decrease in TBW, ICW and ECW).	96.15
Ramos-Vázquez AG. [30]	Neurodegenerative, stroke, head and neck, autoimmune disease, infectious disease, other.	EAT-10 and V-VST	PA°	79 (Nectar = 27 Spoon-thick = 27 Exclusive tube feeding = 25)	More severe OD patients showed a lower PA°, which was related to an alteration of cell integrity and permeability.	87.50
**Biochemical studies**						
Lee A. [42]	Geriatric	Bedside swallowing test and/or SLP	Not specified	211	Patients with swallowing impairments were at increased risk of dehydration.	72.22
Churchill M. [36]	Stroke	BDST, MBS	BUN/Cr ratio, BUN, serum sodium	296	Dysphagia was a marker for increased risk of dehydration.	85.00
Crary MA. [31]	Stroke	MASA, FOIS	BUN/Cr ratio	67	Ischemic stroke patients with OD were at risk for dehydration on admission to the hospital.	72.73
Crary MA. [13]	Stroke	MASA, FOIS	BUN/Cr ratio	64	Poor hydration status among acute ischemic stroke with a decrease in hydration specific to patients with dysphagia.	60.00
Schmidt J. [37]	Stroke	MBS technique	Serum sodium, BUN	59	No significant differences in dehydration were observed between aspirators and non-aspirators.	40.00
Smithard DG. [41]	Stroke	VFS	Hematocrit, plasma sodium, urea and osmolality	121	Patients with swallowing difficulties were more likely to use parenteral fluids (*p* < 0.001) and for longer times (*p* < 0.0001).	68.18
Botigué T. [32]	Geriatric	V-VST	BUN/Cr ratio, blood osmolarity, serum sodium	53	No significant differences were observed in hydration status between OD and non-OD patients.	75.00
Buoite SA. [39]	Acute stroke	V-VST	Urine osmolality	95 (OD = 18 nOD = 56 Not evaluated = 21)	OD was not significantly associated with a higher risk of dehydration on discharge.	90.00
Murray J. [35]	Stroke	VFS	BUN/Cr ratio	100	Dysphagia was not a significant predictor of any of the outcomes measured.	72.73
Goroff H. [34]	Stroke	Previous records. If needed FEES or VFS.	BUN/Cr ratio, BUN, serum sodium	712 (Liquid = 675 Nectar or honey = 33 Honey = 4)	Mild dehydration on discharge from the acute care hospital.	90.90
Sala R. [40]	CVA	Standardized test of dysphagia	Urea	187	Mild dehydration in the whole group demonstrated by increased serum urea.	81.80
Kim KL. [43]	Geriatric	VFS	BUN/Cr ratio	52	No significant differences were observed in the BUN/Cr ratio between the two groups.	65.00
Leibovitz A. [14]	Geriatric	FOSS (SLP evaluation)	BUN/Cr ratio, BUN, serum osmolarity, urine sodium, urine osmolality, serum creatinine, serum sodium, urine/creatinine, urine/serum osmolality	95	The mean number of dehydration markers was significantly higher in the FOSS-2 group compared with NGT-fed patients.	87.50
Howard MM. [38]	CVA, TBI	FOIS, PenAsp Scale, MBS	BUN, creatinine, serum sodium	20 (CVA = 19 TBI = 1)	BUN, creatinine and serum sodium levels were high, indicating signs of dehydration in the initial stage of the study.	63.64
Murray J. [35]	Stroke	VFS	BUN/Cr ratio	14	Most participants were classified as dehydrated on entry to the study.	80.77

^1^ QA, Quality assessment: a higher score indicates a lower risk of bias. OD, oropharyngeal dysphagia; VFS, videofluoroscopy; V-VST, Volume-Viscosity Swallowing Test; ICW, intracellular water; ECW, extracellular water; TBW, total body water; NRL, neurological; CAP, community-acquired pneumonia; PA°, phase angle; SLP, speech language pathologist; FOIS, Functional Oral Intake Scale; EAT-10, Eating Assessment Tool-10; MDADI, MD Anderson Dysphagia Inventory; FOSS, Functional Outcome Swallowing Score; MBS, modified videofluoroscopic barium swallow; BUN, blood urea nitrogen; MASA, Mann Assessment of Swallowing Ability; BUN/Cr, blood urea nitrogen/creatinine; FEES, Fiberoptic Endoscopic Evaluation of Swallowing; CVA, cerebrovascular accident; BDST, Burke Dysphagia Screening Test; NGT, nasogastric tube; PenAsp, penetration–aspiration; TBI, traumatic brain injury.

**Table 2:** Main characteristics and quality assessment in Systematic Review 2.

Study	Study Design	OD Etiology	Assessment for OD	Number of Participants	TF or Product Therapy	Effect on Hydration Status	QA (%) ^1^
Bioimpedance Studies							
Sezgin B. [29]	RCT	Total maxillectomy	FOSS, FOIS, EAT-10, MDADE	12	Use of xanthan-gum for 3 months post-total maxillectomy	Using xanthan-gum-based liquid thickener helped maintain ICW, ECW and TBW.	96.15
**Biochemical studies**							
Goroff H. [34]	Cohort	Ischemic stroke	Previous records; if needed, FEES or VFS.	712 (Safe swallow = 675 Nectar = 33 Honey = 4)	Modified cornstarch and maltodextrin	After an active hydration intervention, there was an improvement in hydration on discharge and a decrease in the need for intravenous hydration.	90.90
DePippo KL. [48]	RCT	Stroke	BDST and MBS	115	3 groups: (A = formal intervention B = formal intervention + reevaluation C = active intervention) TP not specified	Intensity of the treatment (diet alteration and compensatory swallowing techniques) did not affect the development of post-stroke complications.	88.45
Whelan K. [47]	RCT	Acute stroke	SLP or VFS	24	Powder thickened (maize starch) Pre-thickened drink	The results of the study showed no correlation between the traditional biochemical markers of hydration and daily fluid intake or fluid balance.	80.77
Murray J. [35]	RCT	Stroke	VFS	14 (G1:6 G2:8)	Xanthan-gum	Those who were permitted water had improved hydration levels compared to those on TF alone, suggesting even a small amount of water per day made a difference to hydration levels.	80.77
Crary M. [13]	Case-control	Ischemic stroke	MASA, FOIS	64 (38% OD)	TP not specified	Any modification of regular liquids and solid diets contributed to reduced hydration on discharge.	60.00
Howard MM. [38]	Cohort	CVA and TBI	FOIS and PenAsp Scale	20	Pre-packaged TF (starch-based)	Patients receiving a higher viscosity fluid had poorer hydration status compared to those receiving a thin textured fluid.	63.64

OD, oropharyngeal dysphagia; FOSS, Functional Outcome Swallowing Score; FOIS, Functional Oral Intake Scale; EAT-10, Eating Assessment Tool-10; MDADI, MD Anderson Dysphagia Inventory; ICW, intracellular water; ECW, extracellular water; TBW, total body water; RCT, randomized controlled trial; MASA, Mann Assessment of Swallowing Ability; TF, thickened fluid; TP, thickening product; FEES, Fiberoptic Endoscopic Evaluation of Swallowing; VFS, videofluoroscopy; BDST, Burke Dysphagia Screening Test; MBS, modified videofluoroscopic barium swallow; SLP, speech language pathologist; PenAsp, penetration–aspiration. ^1^ QA, Quality assessment: a higher score indicates a lower risk of bias.

**Table 3:** Number of participants studied, quality assessment, and answers to PICO questions for SR-2.

Study	Number of Participants Studied	QA (%) ^1^	PICO QUESTIONS		
			Effect of TF Therapy on Fluid Intake and Hydration Status	TF Therapy Depended on OD Severity or the Type of Thickening Product Used	TF Improved or Aggravated Dehydration
Goroff H. [34]	712	90.90	POSITIVE EFFECT	NOT ANSWERED	POSITIVE EFFECT
Sezgin B. [29]	22	96.15	POSITIVE EFFECT	NOT ANSWERED	POSITIVE EFFECT
DePippo K. [48]	115	88.45	NEUTRAL	NOT ANSWERED	NEUTRAL
Murray J. [35]	14	80.77	NEUTRAL	NOT ANSWERED	NEUTRAL
Whelan K. [47]	24	80.77	NEUTRAL	NOT ANSWERED	NEUTRAL
Crary M. [13]	64	60.00	NEGATIVE EFFECT	NOT ANSWERED	NEGATIVE EFFECT
Howard MM. [38]	20	65.00	NEGATIVE EFFECT	NOT ANSWERED	NEGATIVE EFFECT

^1^ QA, Quality assessment: a higher score indicates a lower risk of bias. TF, thickened fluid.

## Data Availability

The articles included in SR and ScR were obtained from the following databases: PubMed, Embase and Web of Science.

## Supplementary Materials

The following supporting information can be downloaded at: https://www.mdpi.com/article/10.3390/nu14122497/s1, Table S1. Search terms and Mesh terms used in the literature search for R1; Table S2. Search terms and Mesh terms used in the literature search for R2; Supplementary Table S3: Main characteristics and conclusions of studies included in the ScR-2; Supplementary File S2: Risk of bias [81,82,83,84,85,86,87,88,89].
