# Supplementary material for: The Hydration Status of Adult Patients with Oropharyngeal Dysphagia and the Effect of Thickened Fluid Therapy on Fluid Intake and Hydration: Results of Two Parallel Systematic and Scoping Reviews
Source: Nutrients. 2022 Jun 16;14(12):2497. doi: 10.3390/nu14122497 (PMC9228104; doi:10.3390/nu14122497)
Supplement: Supplementary file 1 [file nutrients-14-02497-s001.zip › Supplementary file S1_Protocol.pdf]

The hydration status of adult patients with oropharyngeal dysphagia and the effect of thickened fluid therapy on hydration: protocol for two parallel systematic and scoping reviews

Paula Viñas[1], Mireia Bolívar-Prados[1,2], Noemí Tomsen[1,2], Alicia Costa[1], Sergio Marin[1,3], Stephanie A. Riera[1], Pere Clavé[1,2]

[1] *Gastrointestinal Physiology Laboratory. Hospital de Mataró, Universitat Autònoma de Barcelona, Mataró, Spain*

[2] *Centro de Investigación Biomédica en Red de Enfermedades Hepáticas y Digestivas (CIBERehd), Barcelona, Spain*

[3] *Pharmacy Department, Hospital Universitari Germans Trias i Pujol, Badalona, Catalunya, Spain*

Corresponding Author: Pere Clavé, MD, PhD

Professor of Surgery

Department of Surgery. Hospital de Mataró,

Universitat Autònoma de Barcelona,

Carretera de Cirera s/n, 08304 Mataró, Spain

Tel. +34 93 741 77 00 ext. 1046

E-mail: pere.clave@ciberehd.org

ABSTRACT

Background. Hydropenia and dehydration are highly prevalent among patients with oropharyngeal dysphagia (OD). Thickened fluids (TF) are effective in preventing aspiration in OD. Nevertheless, the relationship between fluid thickening and patient hydration status remains highly controversial. We aim to develop a protocol for two parallel literature reviews summarizing indexed articles that describe the hydration status of patients with OD and/or the effect of TF therapy on fluid intake and hydration status.

Methods/design. We will perform two literature reviews assessing: R1) the hydration status of adult patients affected by OD; and R2) the effect of TF on the hydration status and fluid intake of this population. Each review will include a systematic (SR) and a scoping review (ScR) following PRISMA and PRISMA-ScR methodologies. MEDLINE, Embase and Web of Science will be searched and a subsequent reference check will be done. No publication date or language restriction will be imposed. The main outcomes of interest will be patient hydration status (R1) and the effect of TF on hydration status and fluid intake (R2). Cross-sectional, cohort, case control and randomized studies will be included in the SR in which data will be presented and synthesized using tables and narrative. Case and case-control reports, review articles and other SRs will also be included in the ScR. Quality and strength of evidence assessments will be done using the critical appraisal tools of Joanna Briggs Institute and Grading of Recommendations Assessment, Development and Evaluation (GRADE) recommendations respectively.

Discussion: This protocol for parallel literature reviews will be published in a peer-reviewed journal. This study will be the first step towards establishing the pathophysiological relation between hydration status and OD secondary to several etiologies and the potential effect of TF on hydration status and fluid intake.

Systematic review registration. Both SR protocols have been registered in PROSPERO with code: CRD42021272030 (R1) and CRD42021242098 (R2).

KEYWORDS: Deglutition; Deglutition disorders; Dysphagia; Hydration; Hydropenia; Dehydration; Nutritional status; Aspiration, Thickened fluids, Thickeners

Strengths and limitations of this study

- This systematic and scoping review protocol was performed using the Preferred Reporting Items for Systematic Reviews and Meta-Analyses (PRISMA) recommendations and PRISMA extension for the scoping review.
- The proposed methodology will allow us to assess the quality of the selected studies.
- The bibliographical search considers MEDLINE, Embase and Web of Science databases.
- Unpublished material and abstracts will not be included in any of both reviews.
- The results will be a first step towards establishing the relationship between dehydration and OD and the potential effect of thickened fluids on hydration status and fluid intake.

BACKGROUND

Oropharyngeal dysphagia (OD) is a symptom of the difficulty or inability to form or safely move a bolus from the mouth to the esophagus (1). OD is recognized by the World Health Organisation in the International Classification of Diseases (ICD) with codes 787.2 and R13 for ICD-9 and ICD-10, respectively (2). OD is a prevalent condition among the older population, and has been recognized as a geriatric syndrome by two European societies, European Society for Swallowing Disorders and European Geriatric Medicine Society (3).

Swallowing impairments can cause two main groups of complications in dysphagic patients: a) efficacy impairments leading to malnutrition and dehydration and b) safety impairments leading to tracheobronchial aspirations which can produce respiratory infections and aspiration pneumonia with high mortality rates (4, 5). Although dehydration is considered to be one of the major complications of OD in older people (4), the research attention on this topic has been quite low. Several studies have shown hydropenia and reduction in the intracellular water (ICW) compartment and saliva volume in patients with OD (6,7), which could be the consequence of reduced water intake associated to OD, loss of the sensation of thirst, and also to a loss of body fluids with a lower osmolality with respect to plasma (8). In addition to OD, thickened fluids (TF), a valid therapeutic strategy for patients with OD to reduce the risk of airway invasion (9), may also contribute to lower fluid intake due to low palatability at high viscosity levels (10). TF have been proved to be very effective in acute instrumental clinical trials to prevent aspirations (10-13), and multimodal strategies including TF have shown an improvement in nutritional status and reduction in respiratory infections at 6-month follow-up (14).

Shear viscosity is the main physical property involved in the therapeutic effect of TF (15). Increasing shear viscosity has proved to increase the prevalence of safe swallows in several phenotypes of patients with OD: older, post-stroke (10,13), head and neck cancer and neurodegenerative diseases (13). However, the therapeutic effect of TF depends on several factors such as the composition of the selected thickening product (TP), the preparation method, the amount of TP, the thickness, and the rheological parameters such as salivary amylase in the oral

phase and shear thinning in the pharyngeal phase (15). TP can be divided into two main groups according to their composition: modified starch (MS) based and xanthan gum (XG) based. MS absorbs water and swells, is less stable over time and less resistant to salivary amylase (16). Xanthan gum products form stable bonds with water and present higher resistance to oral amylase. Very few studies have compared the therapeutic effect of both types of TP on the same phenotype of patients like Vilardell et al (16) in post-stroke patients with OD. This study concluded that to provide the same therapeutic effect, a higher viscosity was needed for MS than XG fluids. MS thickener showed significantly higher prevalence of oral and pharyngeal residue at thin liquid-, nectar- and spoon-thick viscosities in comparison with XG. Another concern is the need to express viscosity in mPa·s in these studies, as determined by the International System of Units (SI) (17) and the rules of ICMJ for scientific manuscripts. Also, to describe the effect of α-SA and shear thinning as they are major factors affecting the therapeutic effect and mode of action of thickening agents (18). Another factor, which should be considered when prescribing TP, is palatability as this decreases when fluid viscosity is increased, whether they are made of xanthan gum (10) or modified corn-starch and maltodextrin (19). These aspects have also been related to an increase in OD complications such as dehydration.

Taking all these concerns together, some experts suggest the need for further research to discover the main cause of dehydration in this specific population and a better monitorization of hydration status in those receiving TF (20). The current situation surrounding the hydration status of patients with OD and the effect of TF on this status leaves some questions unanswered, particularly in relation to the risk-benefits of increasing bolus viscosity between improving airway protection and worsening palatability and possibly fluid intake. Our study will aim to assess, summarize and map all literature available on the hydration status of adult patients affected by OD and the effect of TF therapy on the fluid intake and the hydration status on these patients.

METHODS AND DESIGN

We will perform two parallel literature reviews assessing: R1) the hydration status of adult patients affected by OD, and R2) the therapeutic effect of TF therapy on hydration status in this population. Each review will include a systematic (SR) and a scoping review (ScR):

Review 1 (*Hydration status of patients with OD).* We will include cross-sectional, cohort, case control (for instance, studies in which cases were patients with OD and controls no) and randomized studies in which hydration status is assessed using an objective method (such as analytical measurements or bioelectrical impedance analysis (BIA)) for the SR. These and review articles, cases and case-control reports will be included in the ScR along with cross-sectional, cohort, case control and randomized studies in which hydration status is assessed using subjective methods. The main outcome of interest will be the hydration status in adult patients with OD.

Review 2 *(Effect of TF therapy on hydration status).* To answer the question on the potential beneficial or deleterious effect of TF therapy on the hydration status of patients with OD, we will elaborate another SR and ScR, to identify the utility of TF on this condition and also on fluid intake. We will identify, summarize and present the main reported concepts, theories and knowledge gaps in this field. The same type of articles for the SR and ScR will be included as for R1.

1) **Preferred Reporting Items for Systematic Reviews and Meta-Analyses (PRISMA) statement**

SRs are basic tools to assess the evidence related to a topic with precision and trustworthiness. Nowadays, there are specific instruments to update knowledge on a certain topic, achieve conclusions on available evidence and take decisions on healthcare conditions. SRs must thus follow accurate methodology and be reported with transparency. For this reason, we will use the methodology proposed by Preferred Reporting Items for Systematic Reviews and Meta-Analyses (PRISMA) to carry out this SR (21). In this protocol, we have followed the recommendations stated by PRISMA Protocols annex (PRISMA-P) to report protocols for SR. PRISMA-P also offers study examples in the protocol for each item extracted from studies that were relevant in their respective fields, reported with high quality and carried out using accurate methodology (22,23). Protocols are necessary in

order to increase work quality and reduce the risk of bias to the minimum. For all these reasons, we use PRISMA as a reference in this work. PRISMA-P checklist is available in Additional File 1. To complement each SR with a ScR, we will use the PRISMA extension for ScR (PRISMA-ScR) (24).

2) Registration

This SR has been registered in the International Prospective Register of Systematic Reviews of the Center for Reviews and Dissemination (PROSPERO)((https://www.crd.york.ac.uk/prospero/). Registration number: R1 (CRD42021272030) and R2 (CRD42021242098).

3) Literature Search

We will search MEDLINE using PubMed, Embase using OVID and Web of Science. The combined Mesh and search terms used in PubMed searches are described in Table 1 (R1) and Table 2 (R2). Equivalent search strategies will be applied to subsequent searches. The list of references of the selected articles will be checked in order to find additional eligible studies. No publication date or language restrictions will be imposed. Unpublished material, pre-prints, posters, protocols, surveys, book chapters, doctoral thesis and abstracts will not be included.

Table S1. Search terms and Mesh terms used in the literature search for R1.

**Terms related to oropharyngeal dysphagia and connected among themselves by ‘OR’**	**Terms related to hydration status connected among themselves by ‘OR’**

1. "Deglutition Disorders"[Mesh]<br>2. "Deglutition"[Mesh]<br>3. Deglutition*[tiab]<br>4. Swallow*[tiab]<br>5. Dysphag*[tiab]	6. "Dehydration"[Mesh]<br>7. Hydrat*[tiab]<br>8. Dehydrat*[tiab]<br>9. "Organism hydration status"[Mesh]<br>10. "Extracellular fluid"[Mesh]<br>11. "Intracellular fluid"[Mesh]<br>12. "Water-electrolyte balance"[Mesh]<br>13. "Body water"[Mesh]

Terms detailed in the two columns above, related to oropharyngeal dysphagia and hydration status, will be connected using 'AND'.

[a]tiab= Title/Abstract

Subsequently, the following terms will be added using NOT:"Gastroesophageal reflux"[tiab]), and (Gastroesophageal reflux[Mesh].

Table S2. Search terms and Mesh terms used in the literature search for R2.

**Terms related to oropharyngeal dysphagia and connected among themselves by 'OR'**	**Terms related to therapeutic effect and hydration and connected among themselves by 'OR'**	**Terms related to thickeners or thickened fluids or thickened products and connected among themselves by 'OR'**

1. “Deglutition Disorders”[Mesh]<br>2. “Deglutition”[Mesh]<br>3. “Swallow”[Title]<br>4. “Swallowing”[Title]<br>5. Dysphag*[Title]	6. “Therapeutic effects”[tiab]<br>7. “Therapeutic effect”[tiab]<br>8. “Side effects”[tiab]<br>9. “Adverse effects”[tiab]<br>10. “Nutritional Status”[Mesh]<br>11. “Quality of life”[Mesh]<br>12. “Dehydration”[Mesh]<br>13. “Malnutrition”[Mesh]<br>14. “Hydration”[tiab]<br>15. “Nutrition assessment”[Mesh]<br>16. “Organism hydration status”[Mesh]	17. “Food, Formulated”[MAJR]<br>18. “Viscosity”[Mesh]<br>19. “Fluid”[tiab]<br>20. Thicken*[tiab]<br>21. “Fluids”[tiab]<br>22. “Liquids”[tiab]

Terms detailed in the three columns above, related to oropharyngeal dysphagia, thickeners or thickened products and their effect on hydration status, will be connected using ‘AND’.

4) <u>Selection process</u>

We will assess the articles identified through the literature search to select eligible studies. We will use a double phase process that will include an initial screening phase and a subsequent definitive selection phase according to eligibility criteria. In the first screening phase, the abstract and title of the studies will be analyzed to eliminate studies not containing at least minimal relevant information on the hydration status of adult patients affected by OD (R1) or about the effect of TF therapy on

hydration status and fluid intake in these patients (R2). In the abstract or title of articles for R1, there must appear terms related to “dysphagia” or “deglutition” or “swallowing” and “hydration status” or “dehydration” or “hydration”; and in addition, for R2, terms related to “thickeners” or “thickened fluids” or “thickened products”. Duplicates will be removed in this initial phase. This selection process will be done by two reviewers and, in case of disagreement between them, a third reviewer's opinion will be sought to reach a consensus and take a final decision.

In the second selection phase, the complete text of the selected articles will be assessed and they will be included according to eligibility criteria.

Review 1 (*Hydration status of patients with oropharyngeal dysphagia*)

Articles will be included in the SR if they report information on adult patients (≥17 years) affected by OD (confirmed by clinical or instrumental assessment) in which hydration status was measured using an objective method (analytical measurement or BIA). In the case of patients receiving specific intervention for hydration status, only basal hydration will be considered. Articles will be excluded if patient hydration status was not assessed or if they referred to esophageal dysphagia.

The ScR will include articles in which hydration status was measured but in this case with a subjective method was evaluated.

Review 2 (*Effect of TF therapy on hydration status)*

Those articles where the hydration status was assessed objectively by analytical measurement or BIA and the effect of TF therapy was reported in adult patients affected by OD will be included in the SR. Articles will be excluded if patient hydration status was not assessed, if they referred to esophageal dysphagia or if TF were not used.

The ScR will include the articles in which the effect of TF therapy on hydration status or fluid intake was assessed also with subjective methods was evaluated.

Two reviewers will perform this process independently. Subsequently, the articles selected will be compared and a third reviewer will make a final decision for each case of disagreement. The main reasons for article exclusion will be mentioned. In both reviews, no restrictions will be imposed

regarding size of the sample. The selection process is summarized in Figure 1, where three independent reviewers may participate.

Figure 1. PRISMA flowchart showing data selection process for the two parallel reviews assessing the hydration status of patients with OD (Review 1); and the effect of TF therapy on hydration status and fluid intake of dysphagic patients (Review 2).

Data collection and data items

Data from the selected studies will be independently extracted by two reviewers and registered in a standard data collection form. These data will be compared and, in case of disagreement between reviewers, a third reviewer's opinion will be sought to reach a consensus and take a final decision. In case of any unreported information or lack of clarity, authors of included studies will be contacted. Data obtained directly from the authors will be clearly identified. Any lost or unavailable information will be reported. To manage study data, we will transfer all information in the data collection form to a spreadsheet. Data gathering will refer to main study characteristics, quality assessment and study results.

Review 1 (*Hydration status of OD patients)*

The information to be obtained from each study is presented as follows: a) ***Study identification***: first author, journal and year of publication; b) ***Main study characteristics***: type of study, epidemiological approach (cross-sectional or longitudinal), data gathering (retrospective or prospective), time horizon, time of hydration status evaluation, presence of a control group (patients without OD), location/setting; c) ***Sample characteristics***: sample size; sociodemographic data (age, average and range; gender), patient inclusion and exclusion criteria, method of OD assessment or similar, main disease causing OD (if applicable); d) ***Data source*****:** medical registries, Medicare database, national patient databases, insurance database, data collected from individual research groups, data collected from individual hospitals; e) ***Study results*****:** the primary outcome will be the hydration status of patients with OD measured with **objective methods (SR)**: (i) BIA: total body water (TBW), intracellular water, extracellular water (ECW), ECW/TBW ratio, phase angle and impedance ratio

(25,26) (ii) biochemical data: laboratory tests such as blood and urine analytical parameters: blood osmolarity (15, 27), urea nitrogen/creatinine ratio (28), sodium concentration, urine osmolality, urine-specific gravity (29,30); or **subjective methods (ScR):** (i) *signs and symptoms* (reduced urine production, dry mouth, thirst, dry skin, reduced tear production, sunken eyes, obnubilation, drowsiness and perception of fluid intake; other manifestations such as tachycardia, reduced circulation volume, acidosis, potassium depletion, renal damage, intestinal ischemia or heart failure (31) and (ii) *Others such as:* the volume of daily fluid intake (mL); g) ***Quality assessment***: explained later.

Review 2 (*Effect of TF therapy on hydration status)*

The same information as R1 will be obtained but include: a) ***Intervention characteristics***: type of TP used (based on xanthan gum, starch or mixed), viscosity level (by qualitative descriptors or shear viscosity units in mPa·s), volume ml/24h of TP provided/ingested to/by patients; or any patient hydration protocol.

Quality assessment and confidence in cumulative evidence

Quality of studies will be evaluated using the critical appraisal tools provided by the Joanna Briggs Institute (JBI) specific for each type of study included in the SR. JBI checklists provide a set of items to assess trustworthiness, relevance and results of studies to be included in a SR. Each item represents an aspect related to the internal validity of the included studies and will be rated as "Yes, no, unclear or not applicable". For each study, the total amount of items will be rated as "yes (1 point)", "unclear (0.5 points)" and "no (0 points)" and then it will be divided between the total applicable items. This total score will be presented as a percentage in which a higher score will represent a lower risk of bias. No study will be eliminated based on its risk of bias, but we will assess how risk of bias can affect the main results and outcome measures. Strength of the evidence will be evaluated using the Grading of Recommendations Assessment, Development and Evaluation (GRADE) system (32). GRADE is a tool designed to assess the strength of the summarized evidence across studies in SR by evaluation of both study limitations, imprecision, inconsistency of results, indirectness of evidence, publication bias, and magnitude of the effect and the presence of confounders that minimize the effect. Regarding

ScR, authors will not perform the quality assessment of included studies as PRISMA-ScR does not consider it essential (24).

Data presentation and data synthesis

Data presentation and synthesis will be performed according to the information obtained. Main study information will be presented and summarized in several tables of evidence. No conversion of study data will be performed, and all data will be presented in their original format during the initial presentation of the results. Due to the expected heterogeneity in the results, we do not plan to perform a quantitative data synthesis in this study. A systematic meta-narrative synthesis will be performed following the SWiM (Synthesis without meta-analysis) guideline recommendations (33). In this meta-narrative synthesis we will describe both the prevalence of dehydration in patients with OD, the hydration status of patients with OD vs. those without (SR1) and the effect of TF on hydration status and fluid intake of patients with OD (SR2). Results will be presented according to: (1) studies in which hydration status was assessed by biochemical parameters and (2) studies in which hydration status was assessed using BIA (SR1 and SR2). In case of studies reporting data using both biochemical and BIA measurements data will be presented separately. Moreover, information of OD severity status (for instance, tube feeding dependency) and patients' clinical condition (hospitalized or acute care vs. chronic condition) will be considered and information will be ordered according to this circumstances. The SR will be supported by ScR which will include all the available published articles containing data on the hydration status of patients affected by OD and the effect of TF on this condition and the results will be presented in a narrative form.

DISCUSSION

OD and swallowing dysfunction might increase the risk of dehydration in several phenotypes of patients although the prevalence of hydropenia and dehydration has not yet been determined. Clinical research on dehydration as a potential complication of swallowing disorders has received less attention than malnutrition and aspiration pneumonia, probably because of the difficulty in assessing hydropenia and dehydration in dysphagic patients. In addition, many older patients with dysphagia might have other factors contributing to dehydration such as: a) factors causing fluid intake restriction

such as immobility, frailty, cognitive disorders, dementia or depression, and reduced thirst; or b) factors causing excessive losses such as gastrointestinal disorders (diarrhea, vomiting), underlying disease (kidney disease, diabetes, heart failure), and medication (diuretics, blood pressure regulators) among other causes that may lead to dehydration (34). There is also little information on the relationship between the severity of OD and prevalence and severity of dehydration. Dehydration may lead to severe complications in patients with dysphagia, including poor muscle function, lethargy, mental confusion, further increasing aspiration risk (4). In addition, dehydration depresses the immune system, making the patient susceptible to infection, and it may also be a risk factor for pneumonia, as it also decreases salivary flow (thus promoting altered microbial colonization of the oropharynx) (35). Several studies support the use of TF as a valid therapeutic strategy to reduce the risk of airway invasion and other complications caused by OD (9-13, 16, 36, 38); however, it has been hypothesized that TF provided to reduce aspirations in patients with dysphagia might also exacerbate dehydration in these patients. Based on clinical expertise -not in evidence-, O'Keeffe et al. suggested TF interventions may be associated with reduced fluid intake, as well as worsening the quality of life of those with dysphagia (20). A SR from Beck AM. 2017 (37) aimed at providing recommendations for adult patients with OD and therapy for malnutrition, dehydration, aspiration and aspiration pneumonia. She concluded that, based on the quality of the evidence, assessment of the risk benefit ratio, and perceived patient preferences, a weak recommendation against the use of texture modified liquids and good clinical practice pointing to the use of texture modified foods in patients with OD could be made (37). In contrast, a study with a multimodal intervention including fluid thickening with MS and texture modified foods clearly show a positive effect on clinical outcomes of elderly patients including a reduction in the incidence of respiratory infections, and an improvement in nutritional status, QoL and morbimortality (14). Our present study will collect all the available evidence on the effect of TF therapy and TP on the hydration status of patients with dysphagia and of different TP at different shear viscosity levels and their relation to compliance and fluid intake. These reviews are focused on the hydration status of older patients with OD and the effect of TP therapy on this condition. It will include all the studies published to date. It is anticipated that the compilation of

all these results in a unique manuscript will contribute to greater knowledge on management the hydration status of patients with OD and optimal monitorization of the use of TF and TP.

LIST OF ABBREVIATIONS

OD: Oropharyngeal dysphagia

ICD: International Classification of Diseases

TP: Thickening products

TF: Thickened fluids

MS: Modified starch

XG: Xanthan gum

SR: Systematic review

ScR: Scoping review

R1: Review 1

R2: Review 2

PRISMA: Preferred Reporting Items for Systematic Reviews and Meta-Analyses

PROSPERO: International prospective register of systematic reviews

PICO: Population, Intervention, Comparison and Outcome.

BIA: Bioelectrical Impedance Analysis

ICW: Intracellular water

TBW: Total body water

ECW: Extracellular water

GRADE: Grading of Recommendations, Assessment, Development and Evaluation

SWiM: Synthesis without meta-analysis

DECLARATIONS

Patient and public involvement statement

There was no public or patient involvement in the elaboration of this protocol.

Ethics approval and consent to participate

Not applicable

Consent for publication

Not applicable

Availability of data and materials

The articles included in SR and ScR were obtained from the following databases: PubMed, Embase and Web of Science.

Competing interests

PV, MB-P, NT, AC and PC declare that they have received fees from Nestlé Healthscience for carrying out this systematic review.

Funding

This study is funded by an educational grant from Nestle Health Science and Fundació Salut del Consorci Sanitari del Maresme.

Authors' contributions

PC designed the protocol; PV, MB-P and NT are the first, second and third reviewers, respectively; AC provided a critical revision of all the protocol sections; SM and SR provided expertise on investigation methodology and quality assessment; PC, PV, MB-P, NT, AC and SM wrote the article. All authors provided a critical revision and approved the final revision.

Acknowledgements

We would like to thank Agustí Viladot, member of Mataró Hospital library service, for helping with the search strategy. This work has been conducted within the framework of a doctoral thesis in medicine from the Medicine Department of the Autonomous University of Barcelona. The Territorial Competitiveness Specialization Project (PECT) of Mataro-Maresme (PRE/161/2019) financed by the Government of Catalunya-Generalitat de Catalunya within the framework of the European Regional Developments Funds of Catalonia Operational Programme 2014-2020.

REFERENCES

1. Clavé P, Shaker R. Dysphagia: current reality and scope of the problem. Nat Rev Gastroenterol Hepatol. 2015;12(5):259-270.

2. World Health Organization. International Statistical Classification of Diseases and Related Health Problems 10th Revision. 2016.
3. Baijens, L WJ; Clavé, P; Cras, P; Ekberg, O; et al. European society for swallowing disorders - European union geriatric medicine society white paper: Oropharyngeal dysphagia as a geriatric syndrome. Clin Interv Aging. 2016;11:1403–28.
4. Rofes, L; Arreola, V; Romea, M; Palomera, E; et al. Pathophysiology of oropharyngeal dysphagia in the frail elderly. Neurogastroenterol Motil. 2010;22(8):1–9.
5. Serra-Prat, M; Palomera, M; Gomez, C; Sar-Shalom, D; et al. Oropharyngeal dysphagia as a risk factor for malnutrition and lower respiratory tract infection in independently living older persons: A population-based prospective study. Age Ageing. 2012;41(3):376–81.
6. Carrión S, Roca M, Costa A, Arreola V, Ortega O, Palomera E, Serra-Prat M, Cabré M, Clavé P. Nutritional status of older patients with oropharyngeal dysphagia in a chronic versus an acute clinical situation. Clin Nutr. 2017;36(4):1110-1116.
7. Tomsen N, Ortega O, Nascimento W, Carrión S, Clavé P. Oropharyngeal Dysphagia in Older People is Associated with Reduced Pharyngeal Sensitivity and Low Substance P and CGRP Concentration in Saliva. Dysphagia. 2021.
8. Cheuvront SN, Kenefick RW. Dehydration: physiology, assessment, and performance effects. Compr Physiol 2014;4:257e85.
9. Newman R, Vilardell N, Clavé P, Speyer R. Effect of Bolus Viscosity on the Safety and Efficacy of Swallowing and the Kinematics of the Swallow Response in Patients with Oropharyngeal Dysphagia: White Paper by the European Society for Swallowing Disorders (ESSD). Dysphagia. 2016;31(2):232-49.
10. Bolivar-Prados M, Rofes L, Arreola V, Guida S, Nascimento WV, Martin A, Vilardell N, Ortega Fernández O, Ripken D, Lansink M, Clavé P. Effect of a gum-based thickener on the safety of swallowing in patients with poststroke oropharyngeal dysphagia. Neurogastroenterol Motil. 2019;31(11):e13695.

11. Clavé P, de Kraa M, Arreola V, Girvent M, Farré R, Palomera E, Serra-Prat M. The effect of bolus viscosity on swallowing function in neurogenic dysphagia. Aliment Pharmacol Ther. 2006;1;24(9):1385-94.
12. Rofes L, Arreola V, Mukherjee R, Swanson J, Clavé P. The effects of a xanthan gum-based thickener on the swallowing function of patients with dysphagia. Aliment Pharmacol Ther. 2014;39(10):1169-79.
13. Ortega O, Bolívar-Prados M, Arreola V, Nascimento WV, Tomsen N, Gallegos C, Brito-de La Fuente E, Clavé P. Therapeutic Effect, Rheological Properties and α-Amylase Resistance of a New Mixed Starch and Xanthan Gum Thickener on Four Different Phenotypes of Patients with Oropharyngeal Dysphagia. Nutrients. 2020;23;12(6):1873.
14. Martín A, Ortega O, Roca M, Arús M, Clavé P. Effect of A Minimal-Massive Intervention in Hospitalized Older Patients with Oropharyngeal Dysphagia: A Proof of Concept Study. J Nutr Health Aging. 2018;22(6):739-747.
15. Gallegos C, Brito-de la Fuente E, Clavé P, Costa A, Assegehegn G. Nutritional Aspects of Dysphagia Management. Adv Food Nutr Res. 2017;81:271-318.
16. Vilardell N, Rofes L, Arreola V, Speyer R, Clavé P. A Comparative Study Between Modified Starch and Xanthan Gum Thickeners in Post-Stroke Oropharyngeal Dysphagia. Dysphagia. 2016.
17. BOE-A-2014-13359 Ley 32/2014, de 22 de diciembre, de Metrología. Gobierno de España.
18. Bolívar-Prados M, Tomsen N, Arenas C, Ibáñez L, Clavé P. A bit thick: Hidden risks in thickening products' labelling for dysphagia treatment. Food Hydrocoll. 2021;14.
19. Yver CM, Kennedy WP, Mirza N. Taste acceptability of thickening agents. World J Otorhinolaryngol Head Neck Surg. 2018;21;4(2):145-147.
20. O'Keeffe ST. Use of modified diets to prevent aspiration in oropharyngeal dysphagia: is current practice justified? BMC Geriatr. 2018;20;18(1):167.

21. Liberati A, Altman DG, Tetzlaff J, Mulrow C, Gøtzsche PC, Ioannidis JPA, et al. The PRISMA statement for reporting systematic reviews and meta-analyses of studies that evaluate healthcare interventions: explanation and elaboration. BMJ. 2009;339:b2700.
22. Shamseer L, Moher D, Clarke M, Ghersi D, Liberati A, Petticrew M, Shekelle P, Stewart LA; PRISMA-P Group. Preferred reporting items for systematic review and meta-analysis protocols (PRISMA-P) 2015: elaboration and explanation. BMJ. 2015;2;350:g7647.
23. Moher D, Shamseer L, Clarke M, Ghersi D, Liberati A, Petticrew M, Shekelle P, Stewart LA. Preferred Reporting Items for Systematic Review and Meta-Analysis Protocols (PRISMA-P) 2015 statement. Syst Rev. 2015;4(1):1.
24. Tricco AC, Lillie E, Zarin W, O'Brien KK, Colquhoun H, Levac D, et al. PRISMA Extension for Scoping Reviews (PRISMAScR): Checklist and Explanation. Ann Intern Med. 2018;169:467–473.
25. Seoane F, Abtahi S, Abtahi F, Ellegård L, Johannsson G, Bosaeus I, et al.Mean expected error in prediction of Total body water: a true accuracy comparison between bioimpedance spectroscopy and single frequency regression equations. Biomed Res Int. 2015.
26. Mulasi U, Kuchnia AJ, Cole AJ, Earthman CP. Bioimpedance at the bedside current applications, limitations, and opportunities. Nutr Clin Pract. 2015;30(2):180–93.
27. Plaisier A, Maingay-de Groof F, Mast-Harwig R, Kalkman PM, Wulkan RW, Verwers R, Neele M, Hop WC, Groeneweg M. Plasma water as a diagnostic tool in the assessment of dehydration in children with acute gastroenteritis. Eur J Pediatr. 2010;169(7):883-6.
28. Yilmaz K, Karaböcüoglu M, Citak A, Uzel N. Evaluation of laboratory tests in dehydrated children with acute gastroenteritis. J Paediatr Child Health. 2002;38(3):226-8.
29. Çelik T, Altekin E, İşgüder R, Kenesari Y, Duman M, Arslan N. Evaluation of neutrophil gelatinase-associated lipocalin in pediatric patients with acute rotavirus gastroenteritis and dehydration. Ital J Pediatr. 2013;3;39:52.
30. Levy JA, Waltzman M, Monuteaux MC, Bachur RG. Value of point-of-care ketones in assessing dehydration and acidosis in children with gastroenteritis. Acad Emerg Med. 2013;20(11):1146-50.

31. Lacey J, Corbett J, Forni L, Hooper L, Hughes F, Minto G, Moss C, Price S, Whyte G, Woodcock T, Mythen M, Montgomery H. A multidisciplinary consensus on dehydration: definitions, diagnostic methods and clinical implications. Ann Med. 2019;51(3-4):232-251.
32. Guyatt G, Oxman AD, Akl EA, et al. . GRADE guidelines: 1. Introduction-GRADE evidence profiles and summary of findings tables. J Clin Epidemiol 2011;64:383–94.
33. Campbell M, McKenzie JE, Sowden A, Katikireddi SV, Brennan SE, Ellis S, Hartmann-Boyce J, Ryan R, Shepperd S, Thomas J, Welch V, Thomson H. Synthesis without meta-analysis (SWiM) in systematic reviews: reporting guideline. BMJ. 2020; 16;368:l6890.
34. Stanga Z., Aubry E. Dehydration in Dysphagia. In: Ekberg O., editor. Dysphagia: Diagnosis and Treatment. Springer International Publishing; Cham, Switzerland: 2019;859–871.
35. Reber E, Gomes F, Dähn IA, Vasiloglou MF, Stanga Z. Management of Dehydration in Patients Suffering Swallowing Difficulties. J Clin Med. 2019;8;8(11):1923.
36. Leonard RJ, White C, McKenzie S, Belafsky PC. Effects of bolus rheology on aspiration in patients with Dysphagia. J Acad Nutr Diet. 2014;114(4):590-4.
37. Beck AM, Kjaersgaard A, Hansen T, Poulsen I. Systematic review and evidence based recommendations on texture modified foods and thickened liquids for adults (above 17 years) with oropharyngeal dysphagia - An updated clinical guideline. Clin Nutr. 2018;37(6 Pt A):1980-1991.
38. Steele CM, Alsanei WA, Ayanikalath S, Barbon CE, Chen J, Cichero JA, Coutts K, Dantas RO, Duivestein J, Giosa L, Hanson B, Lam P, Lecko C, Leigh C, Nagy A, Namasivayam AM, Nascimento WV, Odendaal I, Smith CH, Wang H. The influence of food texture and liquid consistency modification on swallowing physiology and function: a systematic review. Dysphagia. 2015;30(1):2-26.
