# Supplementary material for: The Hydration Status of Adult Patients with Oropharyngeal Dysphagia and the Effect of Thickened Fluid Therapy on Fluid Intake and Hydration: Results of Two Parallel Systematic and Scoping Reviews
Source: Nutrients. 2022 Jun 16;14(12):2497. doi: 10.3390/nu14122497 (PMC9228104; doi:10.3390/nu14122497)
Supplement: Supplementary file 1 [file nutrients-14-02497-s001.zip › Supplementary file S2_Quality and risk of bias.pdf]

JBI CRITICAL APPRAISAL CHECKLIST FOR ANALYTICAL CROSS SECTIONAL STUDIES

Reviewer______PAULA VIÑAS BEUMALA________Date_26/07/2021

Author___Teresa Botigué__Year_2018 Record Number_________

	Yes	No	Unclear	Not applicable
1. Were the criteria for inclusion in the sample clearly defined?	☐	☐ (highlighted)	☐	☐
2. Were the study subjects and the setting described in detail?	☐ (highlighted)	☐	☐	☐
3. Was the exposure measured in a valid and reliable way?	☐ (highlighted)	☐	☐	☐
4. Were objective, standard criteria used for measurement of the condition?	☐ (highlighted)	☐	☐	☐
5. Were confounding factors identified?	☐	☐	☐ (highlighted)	☐
6. Were strategies to deal with confounding factors stated?	☐	☐	☐ (highlighted)	☐
7. Were the outcomes measured in a valid and reliable way?	☐ (highlighted)	☐	☐	☐
8. Was appropriate statistical analysis used?	☐ (highlighted)	☐	☐	☐

Overall appraisal: Include ☐ (highlighted) Exclude ☐ Seek further info ☐

Comments (Including reason for exclusion)

____
____
____
____

JBI CRITICAL APPRAISAL CHECKLIST FOR COHORT STUDIES

Reviewer_______PAULA VIÑAS_____________Date__________26/07/2021

Author____ALEX BUOITE STELLA_____________________Year___2019_ Record Number_________

	Yes	No	Unclear	Not applicable
1. Were the two groups similar and recruited from the same population?	☒	☐	☐	☐
2. Were the exposures measured similarly to assign people to both exposed and unexposed groups?	☒	☐	☐	☐
3. Was the exposure measured in a valid and reliable way?	☒	☐	☐	☐
4. Were confounding factors identified?	☐	☐	☒	☐
5. Were strategies to deal with confounding factors stated?	☐	☐	☒	☐
6. Were the groups/participants free of the outcome at the start of the study (or at the moment of exposure)?	☐	☐	☐	☒
7. Were the outcomes measured in a valid and reliable way?	☒	☐	☐	☐
8. Was the follow up time reported and sufficient to be long enough for outcomes to occur?	☒	☐	☐	☐
9. Was follow up complete, and if not, were the reasons to loss to follow up described and explored?	☒	☐	☐	☐
10. Were strategies to address incomplete follow up utilized?	☒	☐	☐	☐
11. Was appropriate statistical analysis used?	☒	☐	☐	☐

Overall appraisal: Include ☒ Exclude ☐ Seek further info ☐

JBI CRITICAL APPRAISAL CHECKLIST FOR ANALYTICAL CROSS SECTIONAL STUDIES

Reviewer_____TOMSEN N____Date____28/07/2021

Author______CARRIÓN S. Year 2016   Record Number__________

	Yes	No	Unclear	Not applicable
1. Were the criteria for inclusion in the sample clearly defined?	==☐==	☐	☐	☐
2. Were the study subjects and the setting described in detail?	==☐==	☐	☐	☐
3. Was the exposure measured in a valid and reliable way?	==☐==	☐	☐	☐
4. Were objective, standard criteria used for measurement of the condition?	==☐==	☐	☐	☐
5. Were confounding factors identified?	☐	==☐==	☐	☐
6. Were strategies to deal with confounding factors stated?	☐	==☐==	☐	☐
7. Were the outcomes measured in a valid and reliable way?	==☐==	☐	☐	☐
8. Was appropriate statistical analysis used?	==☐==	☐	☐	☐

Overall appraisal: Include ==☐== Exclude ☐ Seek further info ☐

JBI CRITICAL APPRAISAL CHECKLIST FOR ANALYTICAL CROSS SECTIONAL STUDIES

Reviewer_TOMSEN N._ Date_______28/07/2021_______

Author_____churchill___Year____2004_____ Record Number_________

	Yes	No	Unclear	Not applicable
1. Were the criteria for inclusion in the sample clearly defined?	==☐==	☐	☐	☐
2. Were the study subjects and the setting described in detail?	==☐==	☐	☐	☐
3. Was the exposure measured in a valid and reliable way?	==☐==	☐	☐	☐
4. Were objective, standard criteria used for measurement of the condition?	==☐==	☐	☐	☐
5. Were confounding factors identified?	☐	==☐==	☐	☐
6. Were strategies to deal with confounding factors stated?	☐	==☐==	☐	☐
7. Were the outcomes measured in a valid and reliable way?	==☐==	☐	☐	☐
8. Was appropriate statistical analysis used?	==☐==	☐	☐	☐

Overall appraisal: Include ==☐== Exclude ☐ Seek further info ☐

JBI CRITICAL APPRAISAL CHECKLIST FOR COHORT STUDIES

Reviewer_______PAULA VIÑAS______________Date__________26/07/2021

Author____CRARY M__________________Year___2012_ Record Number_________

	Yes	No	Unclear	Not applicable
1. Were the two groups similar and recruited from the same population?	☐ (highlighted)	☐	☐	☐
2. Were the exposures measured similarly to assign people to both exposed and unexposed groups?	☐ (highlighted)	☐	☐	☐
3. Was the exposure measured in a valid and reliable way?	☐ (highlighted)	☐	☐	☐
4. Were confounding factors identified?	☐	☐	☐ (highlighted)	☐
5. Were strategies to deal with confounding factors stated?	☐	☐	☐ (highlighted)	☐
6. Were the groups/participants free of the outcome at the start of the study (or at the moment of exposure)?	☐	☐	☐ (highlighted)	☐
7. Were the outcomes measured in a valid and reliable way?	☐ (highlighted)	☐	☐	☐
8. Was the follow up time reported and sufficient to be long enough for outcomes to occur?	☐	☐	☐ (highlighted)	☐
9. Was follow up complete, and if not, were the reasons to loss to follow up described and explored?	☐	☐	☐ (highlighted)	☐
10. Were strategies to address incomplete follow up utilized?	☐	☐	☐ (highlighted)	☐
11. Was appropriate statistical analysis used?	☐ (highlighted)	☐	☐	☐

Overall appraisal: Include ☐ (highlighted) Exclude ☐ Seek further info ☐

JBI CRITICAL APPRAISAL CHECKLIST FOR CASE CONTROL STUDIES

Reviewer_____N TOMSEN____Date______28/07/2021_______

Author____CRARY_______Year__2015   Record Number_________

		Yes	No	Unclear	Not applicable
1.	Were the groups comparable other than the presence of disease in cases or the absence of disease in controls?	☒	☐	☐	☐
2.	Were cases and controls matched appropriately?	☒	☐	☐	☐
3.	Were the same criteria used for identification of cases and controls?	☒	☐	☐	☐
4.	Was exposure measured in a standard, valid and reliable way?	☐	☒	☐	☐
5.	Was exposure measured in the same way for cases and controls?	☒	☐	☐	☐
6.	Were confounding factors identified?	☐	☒	☐	☐
7.	Were strategies to deal with confounding factors stated?	☐	☒	☐	☐
8.	Were outcomes assessed in a standard, valid and reliable way for cases and controls?	☐	☒	☐	☐
9.	Was the exposure period of interest long enough to be meaningful?	☒	☐	☐	☐
10.	Was appropriate statistical analysis used?	☒	☐	☐	☐

Overall appraisal: Include ☒ Exclude ☐ Seek further info ☐

JBI CRITICAL APPRAISAL CHECKLIST FOR CASE CONTROL STUDIES

Reviewer______PAULA VIÑAS BEUMALA____Date____26/07/2021_

Author__LYNETTE R. GOLDBERG_____Year_2014 Record Number_________

	Yes	No	Unclear	Not applicable
1. Were the groups comparable other than the presence of disease in cases or the absence of disease in controls?	☑	☐	☐	☐
2. Were cases and controls matched appropriately?	☑	☐	☐	☐
3. Were the same criteria used for identification of cases and controls?	☑	☐	☐	☐
4. Was exposure measured in a standard, valid and reliable way?	☑	☐	☐	☐
5. Was exposure measured in the same way for cases and controls?	☑	☐	☐	☐
6. Were confounding factors identified?	☐	☐	☑	☐
7. Were strategies to deal with confounding factors stated?	☐	☐	☑	☐
8. Were outcomes assessed in a standard, valid and reliable way for cases and controls?	☑	☐	☐	☐
9. Was the exposure period of interest long enough to be meaningful?	☐	☑	☐	☐
10. Was appropriate statistical analysis used?	☑	☐	☐	☐

Overall appraisal: Include ☑ Exclude ☐ Seek further info ☐

JBI CRITICAL APPRAISAL CHECKLIST FOR COHORT STUDIES

Reviewer________PAULA VIÑAS______________Date__________26/07/2021

Author____Goroff____________________Year___2018_ Record Number_________

	Yes	No	Unclear	Not applicable
1. Were the two groups similar and recruited from the same population?	**☐**	☐	☐	☐
2. Were the exposures measured similarly to assign people to both exposed and unexposed groups?	**☐**	☐	☐	☐
3. Was the exposure measured in a valid and reliable way?	**☐**	☐	☐	☐
4. Were confounding factors identified?	**☐**	☐	☐	☐
5. Were strategies to deal with confounding factors stated?	☐	☐	**☐**	☐
6. Were the groups/participants free of the outcome at the start of the study (or at the moment of exposure)?	☐	☐	**☐**	☐
7. Were the outcomes measured in a valid and reliable way?	**☐**	☐	☐	☐
8. Was the follow up time reported and sufficient to be long enough for outcomes to occur?	**☐**	☐	☐	☐
9. Was follow up complete, and if not, were the reasons to loss to follow up described and explored?	**☐**	☐	☐	☐
10. Were strategies to address incomplete follow up utilized?	**☐**	☐	☐	☐
11. Was appropriate statistical analysis used?	**☐**	☐	☐	☐

Overall appraisal: Include **☐** Exclude ☐ Seek further info ☐

JBI CRITICAL APPRAISAL CHECKLIST FOR COHORT STUDIES

Reviewer________PAULA VIÑAS________________Date__________26/07/2021

Author____HOWARD__________________Year___2018_ Record Number_________

	Yes	No	Unclear	Not applicable
1. Were the two groups similar and recruited from the same population?	☐ (highlighted)	☐	☐	☐
2. Were the exposures measured similarly to assign people to both exposed and unexposed groups?	☐ (highlighted)	☐	☐	☐
3. Was the exposure measured in a valid and reliable way?	☐ (highlighted)	☐	☐	☐
4. Were confounding factors identified?	☐	☐ (highlighted)	☐	☐
5. Were strategies to deal with confounding factors stated?	☐	☐ (highlighted)	☐	☐
6. Were the groups/participants free of the outcome at the start of the study (or at the moment of exposure)?	☐	☐	☐ (highlighted)	☐
7. Were the outcomes measured in a valid and reliable way?	☐ (highlighted)	☐	☐	☐
8. Was the follow up time reported and sufficient to be long enough for outcomes to occur?	☐ (highlighted)	☐	☐	☐
9. Was follow up complete, and if not, were the reasons to loss to follow up described and explored?	☐	☐	☐	☐ (highlighted)
10. Were strategies to address incomplete follow up utilized?	☐	☐	☐	☐ (highlighted)
11. Was appropriate statistical analysis used?	☐ (highlighted)	☐	☐	☐

Overall appraisal: Include ☐ (highlighted) Exclude ☐ Seek further info ☐

JBI CRITICAL APPRAISAL CHECKLIST FOR RANDOMIZED CONTROLLED TRIALS

Reviewer_________VIÑAS PAULA_________Date___26/07/2021___

Author_____KL PIPPO___Year 1994 Record Number__________

		Yes	No	Unclear	NA
1.	Was true randomization used for assignment of participants to treatment groups?	**☐**	☐	☐	☐
2.	Was allocation to treatment groups concealed?	**☐**	☐	☐	☐
3.	Were treatment groups similar at the baseline?	**☐**	☐	☐	☐
4.	Were participants blind to treatment assignment?	☐	☐	**☐**	☐
5.	Were those delivering treatment blind to treatment assignment?	☐	☐	**☐**	☐
6.	Were outcomes assessors blind to treatment assignment?	☐	☐	**☐**	☐
7.	Were treatment groups treated identically other than the intervention of interest?	**☐**	☐	☐	☐
8.	Was follow up complete and if not, were differences between groups in terms of their follow up adequately described and analyzed?	**☐**	☐	☐	☐
9.	Were participants analyzed in the groups to which they were randomized?	**☐**	☐	☐	☐
10.	Were outcomes measured in the same way for treatment groups?	**☐**	☐	☐	☐
11.	Were outcomes measured in a reliable way?	**☐**	☐	☐	☐
12.	Was appropriate statistical analysis used?	**☐**	☐	☐	☐
13.	Was the trial design appropriate, and any deviations from the standard RCT design (individual randomization, parallel groups) accounted for in the conduct and analysis of the trial?	**☐**	☐	☐	☐

Overall appraisal: Include **☐** Exclude ☐ Seek further info ☐

JBI CRITICAL APPRAISAL CHECKLIST FOR COHORT STUDIES

Reviewer________PAULA VIÑAS_________________Date___________25/08/2021

Author______LEE______________________Year___1998_ Record Number_________

	Yes	No	Unclear	Not applicable
1. Were the two groups similar and recruited from the same population?	☒	☐	☐	☐
2. Were the exposures measured similarly to assign people to both exposed and unexposed groups?	☒	☐	☐	☐
3. Was the exposure measured in a valid and reliable way?	☒	☐	☐	☐
4. Were confounding factors identified?	☐	☒	☐	☐
5. Were strategies to deal with confounding factors stated?	☐	☒	☐	☐
6. Were the groups/participants free of the outcome at the start of the study (or at the moment of exposure)?	☐	☐	☒	☐
7. Were the outcomes measured in a valid and reliable way?	☒	☐	☐	☐
8. Was the follow up time reported and sufficient to be long enough for outcomes to occur?	☒	☐	☐	☐
9. Was follow up complete, and if not, were the reasons to loss to follow up described and explored?	☐	☐	☐	☒
10. Were strategies to address incomplete follow up utilized?	☐	☐	☐	☒
11. Was appropriate statistical analysis used?	☒	☐	☐	☐

Overall appraisal: Include ☒ Exclude ☐ Seek further info ☐

JBI CRITICAL APPRAISAL CHECKLIST FOR COHORT STUDIES

Reviewer________PAULA VIÑAS______________Date__________26/07/2021

Author____LEIBOVITZ ________________Year___2007_ Record Number_________

	Yes	No	Unclear	Not applicable
1. Were the two groups similar and recruited from the same population?	☒	☐	☐	☐
2. Were the exposures measured similarly to assign people to both exposed and unexposed groups?	☒	☐	☐	☐
3. Was the exposure measured in a valid and reliable way?	☒	☐	☐	☐
4. Were confounding factors identified?	☐	☒	☐	☐
5. Were strategies to deal with confounding factors stated?	☐	☒	☐	☐
6. Were the groups/participants free of the outcome at the start of the study (or at the moment of exposure)?	☐	☐	☐	☒
7. Were the outcomes measured in a valid and reliable way?	☒	☐	☐	☐
8. Was the follow up time reported and sufficient to be long enough for outcomes to occur?	☒	☐	☐	☐
9. Was follow up complete, and if not, were the reasons to loss to follow up described and explored?	☐	☐	☐	☒
10. Were strategies to address incomplete follow up utilized?	☐	☐	☐	☒
11. Was appropriate statistical analysis used?	☒	☐	☐	☐

Overall appraisal: Include ☒ Exclude ☐ Seek further info ☐

JBI CRITICAL APPRAISAL CHECKLIST FOR CASE CONTROL STUDIES

Reviewer TOMSEN N Date____28/07/2021___

Author__LIP K Year_2020 Record Number_________

	Yes	No	Unclear	Not applicable
1. Were the groups comparable other than the presence of disease in cases or the absence of disease in controls?	☐	☑	☐	☐
2. Were cases and controls matched appropriately?	☑	☐	☐	☐
3. Were the same criteria used for identification of cases and controls?	☑	☐	☐	☐
4. Was exposure measured in a standard, valid and reliable way?	☑	☐	☐	☐
5. Was exposure measured in the same way for cases and controls?	☑	☐	☐	☐
6. Were confounding factors identified?	☐	☑	☐	☐
7. Were strategies to deal with confounding factors stated?	☐	☑	☐	☐
8. Were outcomes assessed in a standard, valid and reliable way for cases and controls?	☑	☐	☐	☐
9. Was the exposure period of interest long enough to be meaningful?	☐	☐	☐	☑
10. Was appropriate statistical analysis used?	☑	☐	☐	☐

Overall appraisal: Include ☑ Exclude ☐ Seek further info ☐

JBI CRITICAL APPRAISAL CHECKLIST FOR COHORT STUDIES

Reviewer________PAULA VIÑAS_______________Date__________26/07/2021

Author____MURRAY_______________Year___2018_ Record Number_________

	Yes	No	Unclear	Not applicable
1. Were the two groups similar and recruited from the same population?	☒	☐	☐	☐
2. Were the exposures measured similarly to assign people to both exposed and unexposed groups?	☒	☐	☐	☐
3. Was the exposure measured in a valid and reliable way?	☒	☐	☐	☐
4. Were confounding factors identified?	☒	☐	☐	☐
5. Were strategies to deal with confounding factors stated?	☐	☒	☐	☐
6. Were the groups/participants free of the outcome at the start of the study (or at the moment of exposure)?	☐	☐	☒	☐
7. Were the outcomes measured in a valid and reliable way?	☒	☐	☐	☐
8. Was the follow up time reported and sufficient to be long enough for outcomes to occur?	☐	☐	☒	☐
9. Was follow up complete, and if not, were the reasons to loss to follow up described and explored?	☐	☐	☒	☐
10. Were strategies to address incomplete follow up utilized?	☐	☐	☒	☐
11. Was appropriate statistical analysis used?	☒	☐	☐	☐

Overall appraisal: Include ☒ Exclude ☐ Seek further info ☐

JBI CRITICAL APPRAISAL CHECKLIST FOR RANDOMIZED CONTROLLED TRIALS

Reviewer_____N. TOMSEN_____________Date___25/07/2021____

Author_____MURRAY J___Year 2016 Record Number_________

	Yes	No	Unclear	NA
14. Was true randomization used for assignment of participants to treatment groups?	☐ (highlighted)	☐	☐	☐
15. Was allocation to treatment groups concealed?	☐ (highlighted)	☐	☐	☐
16. Were treatment groups similar at the baseline?	☐ (highlighted)	☐	☐	☐
17. Were participants blind to treatment assignment?	☐	☐ (highlighted)	☐	☐
18. Were those delivering treatment blind to treatment assignment?	☐	☐ (highlighted)	☐	☐
19. Were outcomes assessors blind to treatment assignment?	☐	☐	☐	☐
20. Were treatment groups treated identically other than the intervention of interest?	☐ (highlighted)	☐	☐	☐
21. Was follow up complete and if not, were differences between groups in terms of their follow up adequately described and analyzed?	☐ (highlighted)	☐	☐	☐
22. Were participants analyzed in the groups to which they were randomized?	☐ (highlighted)	☐	☐	☐
23. Were outcomes measured in the same way for treatment groups?	☐ (highlighted)	☐	☐	☐
24. Were outcomes measured in a reliable way?	☐ (highlighted)	☐	☐	☐
25. Was appropriate statistical analysis used?	☐ (highlighted)	☐	☐	☐
26. Was the trial design appropriate, and any deviations from the standard RCT design (individual randomization, parallel groups) accounted for in the conduct and analysis of the trial?	☐ (highlighted)	☐	☐	☐

Overall appraisal: Include ☐ (highlighted) Exclude ☐ Seek further info ☐

JBI CRITICAL APPRAISAL CHECKLIST FOR ANALYTICAL CROSS SECTIONAL STUDIES

Reviewer______PAULA VIÑAS BEUMALA_______Date_26/07/2021

Author___Aniela G Ramos-Vazquez___Year_2020 Record Number_________

	Yes	No	Unclear	Not applicable
1. Were the criteria for inclusion in the sample clearly defined?	☒	☐	☐	☐
2. Were the study subjects and the setting described in detail?	☒	☐	☐	☐
3. Was the exposure measured in a valid and reliable way?	☒	☐	☐	☐
4. Were objective, standard criteria used for measurement of the condition?	☒	☐	☐	☐
5. Were confounding factors identified?	☐	☐	☒	☐
6. Were strategies to deal with confounding factors stated?	☐	☐	☒	☐
7. Were the outcomes measured in a valid and reliable way?	☒	☐	☐	☐
8. Was appropriate statistical analysis used?	☒	☐	☐	☐

Overall appraisal: Include ☒ Exclude ☐ Seek further info ☐

JBI CRITICAL APPRAISAL CHECKLIST FOR COHORT STUDIES

Reviewer________PAULA VIÑAS_______________Date__________25/08/2021

Author_____SALA_____________________Year___1998_ Record Number_________

	Yes	No	Unclear	Not applicable
1. Were the two groups similar and recruited from the same population?	☒	☐	☐	☐
2. Were the exposures measured similarly to assign people to both exposed and unexposed groups?	☒	☐	☐	☐
3. Was the exposure measured in a valid and reliable way?	☒	☐	☐	☐
4. Were confounding factors identified?	☒	☐	☐	☐
5. Were strategies to deal with confounding factors stated?	☐	☐	☒	☐
6. Were the groups/participants free of the outcome at the start of the study (or at the moment of exposure)?	☐	☐	☒	☐
7. Were the outcomes measured in a valid and reliable way?	☒	☐	☐	☐
8. Was the follow up time reported and sufficient to be long enough for outcomes to occur?	☒	☐	☐	☐
9. Was follow up complete, and if not, were the reasons to loss to follow up described and explored?	☐	☐	☒	☐
10. Were strategies to address incomplete follow up utilized?	☐	☐	☒	☐
11. Was appropriate statistical analysis used?	☒	☐	☐	☐

Overall appraisal: Include ☒ Exclude ☐ Seek further info ☐

JBI CRITICAL APPRAISAL CHECKLIST FOR CASE CONTROL STUDIES

Reviewer_____N. TOMSEN____ Date___28/07/2021____

Author____SCHMIDT J__Year 1994 Record Number_________

	Yes	No	Unclear	Not applicable
1. Were the groups comparable other than the presence of disease in cases or the absence of disease in controls?	☐	☒	☐	☐
2. Were cases and controls matched appropriately?	☐	☒	☐	☐
3. Were the same criteria used for identification of cases and controls?	☒	☐	☐	☐
4. Was exposure measured in a standard, valid and reliable way?	☒	☐	☐	☐
5. Was exposure measured in the same way for cases and controls?	☒	☐	☐	☐
6. Were confounding factors identified?	☐	☒	☐	☐
7. Were strategies to deal with confounding factors stated?	☐	☒	☐	☐
8. Were outcomes assessed in a standard, valid and reliable way for cases and controls?	☒	☐	☐	☐
9. Was the exposure period of interest long enough to be meaningful?	☐	☒	☐	☐
10. Was appropriate statistical analysis used?	☐	☒	☐	☐

Overall appraisal: Include ☒ Exclude ☐ Seek further info ☐

JBI CRITICAL APPRAISAL CHECKLIST FOR RANDOMIZED CONTROLLED TRIALS

Reviewer______PAULA VIÑAS______ Date 31/08/2021

Author__BAHA SEZGIN______Year___2018___ Record Number_________

	Yes	No	Unclear	NA
27. Was true randomization used for assignment of participants to treatment groups?	☐ (highlighted)	☐	☐	☐
28. Was allocation to treatment groups concealed?	☐ (highlighted)	☐	☐	☐
29. Were treatment groups similar at the baseline?	☐ (highlighted)	☐	☐	☐
30. Were participants blind to treatment assignment?	☐	☐ (highlighted)	☐	☐
31. Were those delivering treatment blind to treatment assignment?	☐ (highlighted)	☐	☐	☐
32. Were outcomes assessors blind to treatment assignment?	☐ (highlighted)	☐	☐	☐
33. Were treatment groups treated identically other than the intervention of interest?	☐ (highlighted)	☐	☐	☐
34. Was follow up complete and if not, were differences between groups in terms of their follow up adequately described and analyzed?	☐ (highlighted)	☐	☐	☐
35. Were participants analyzed in the groups to which they were randomized?	☐ (highlighted)	☐	☐	☐
36. Were outcomes measured in the same way for treatment groups?	☐ (highlighted)	☐	☐	☐
37. Were outcomes measured in a reliable way?	☐ (highlighted)	☐	☐	☐
38. Was appropriate statistical analysis used?	☐ (highlighted)	☐	☐	☐
39. Was the trial design appropriate, and any deviations from the standard RCT design (individual randomization, parallel groups) accounted for in the conduct and analysis of the trial?	☐ (highlighted)	☐	☐	☐

Overall appraisal: Include ☐ (highlighted) Exclude ☐ Seek further info ☐

JBI CRITICAL APPRAISAL CHECKLIST FOR COHORT STUDIES

Reviewer________PAULA VIÑAS______________Date__________25/08/2021

Author_____SMITHARD_______________________Year___1996_ Record Number_________

	Yes	No	Unclear	Not applicable
1. Were the two groups similar and recruited from the same population?	X			
2. Were the exposures measured similarly to assign people to both exposed and unexposed groups?	X			
3. Was the exposure measured in a valid and reliable way?	X			
4. Were confounding factors identified?		X		
5. Were strategies to deal with confounding factors stated?		X		
6. Were the groups/participants free of the outcome at the start of the study (or at the moment of exposure)?			X	
7. Were the outcomes measured in a valid and reliable way?	X			
8. Was the follow up time reported and sufficient to be long enough for outcomes to occur?	X			
9. Was follow up complete, and if not, were the reasons to loss to follow up described and explored?			X	
10. Were strategies to address incomplete follow up utilized?			X	
11. Was appropriate statistical analysis used?	X			

Overall appraisal: Include [X] Exclude [ ] Seek further info [ ]

JBI CRITICAL APPRAISAL CHECKLIST FOR ANALYTICAL CROSS SECTIONAL STUDIES

Reviewer_ VIÑAS PAULA______Date_____26/07/2021_______

Author_Tomsen_______Year_2021_ Record Number_________

	Yes	No	Unclear	Not applicable
1. Were the criteria for inclusion in the sample clearly defined?	==☐==	☐	☐	☐
2. Were the study subjects and the setting described in detail?	==☐==	☐	☐	☐
3. Was the exposure measured in a valid and reliable way?	==☐==	☐	☐	☐
4. Were objective, standard criteria used for measurement of the condition?	==☐==	☐	☐	☐
5. Were confounding factors identified?	☐	==☐==	☐	☐
6. Were strategies to deal with confounding factors stated?	☐	☐	☐	==☐==
7. Were the outcomes measured in a valid and reliable way?	==☐==	☐	☐	☐
8. Was appropriate statistical analysis used?	==☐==	☐	☐	☐

Overall appraisal: Include ==☐== Exclude ☐ Seek further info ☐

JBI CRITICAL APPRAISAL CHECKLIST FOR RANDOMIZED CONTROLLED TRIALS

Reviewer_____________VIÑAS P_____________ Date__31/08/2021___

Author____WHELAN_______Year 2001 Record Number_________

	Yes	No	Unclear	NA
40. Was true randomization used for assignment of participants to treatment groups?	☒	☐	☐	☐
41. Was allocation to treatment groups concealed?	☐	☐	☒	☐
42. Were treatment groups similar at the baseline?	☒	☐	☐	☐
43. Were participants blind to treatment assignment?	☐	☐	☒	☐
44. Were those delivering treatment blind to treatment assignment?	☐	☒	☐	☐
45. Were outcomes assessors blind to treatment assignment?	☐	☐	☒	☐
46. Were treatment groups treated identically other than the intervention of interest?	☒	☐	☐	☐
47. Was follow up complete and if not, were differences between groups in terms of their follow up adequately described and analyzed?	☒	☐	☐	☐
48. Were participants analyzed in the groups to which they were randomized?	☒	☐	☐	☐
49. Were outcomes measured in the same way for treatment groups?	☒	☐	☐	☐
50. Were outcomes measured in a reliable way?	☒	☐	☐	☐
51. Was appropriate statistical analysis used?	☒	☐	☐	☐
52. Was the trial design appropriate, and any deviations from the standard RCT design (individual randomization, parallel groups) accounted for in the conduct and analysis of the trial?	☒	☐	☐	☐

Overall appraisal: Include ☒ Exclude ☐ Seek further info ☐
