# Supplementary material for: The Hydration Status of Adult Patients with Oropharyngeal Dysphagia and the Effect of Thickened Fluid Therapy on Fluid Intake and Hydration: Results of Two Parallel Systematic and Scoping Reviews
Source: Nutrients. 2022 Jun 16;14(12):2497. doi: 10.3390/nu14122497 (PMC9228104; doi:10.3390/nu14122497)
Supplement: Supplementary file 1 [file nutrients-14-02497-s001.zip › Supplementary Table S3_Results ScR 2.pdf]

Supplementary Table S3. Main characteristics and conclusions of studies included in the ScR-2

Study	Number of participants	Thickened fluid or hydration therapy	Effect on the hydration status
Whelan K 2001	24	Starch (control) and pre-thickened (intervention)	In the stroke population oral intakes of TF are hugely inadequate and indeed no patient was able to achieve their daily fluid requirement through oral intake alone.
Murray J 2016	14	WPG=8<br>TL only=6<br><br>Mildly TL=12<br>Moderately TL=2<br><br>***TP not specified***	Total beverage intake of participants in the WPG was not higher than for those who consumed TL only.<br><br>Even when given the choice to drink water, participants in the water protocol group drank more TL than water.<br><br>Participants in WPG rated their overall satisfaction with water at a similar level to that for TL.
Howard M 2018	20	***Prepackaged thickened liquids from the Thick n Easy product line***. *These pre-thickened liquids are **starch based***	Patients reported when consuming textured thin liquids a significant improvement in their satisfaction related to their thirst.
Karagiannis M 2011	76	Control = 34 only TP<br>Intervention= 42 TP and free access to water<br><br>***TP not specified***	Much higher levels of satisfaction with drinks, level of thirst and mouth cleanliness in the intervention group.<br><br>Patients with access to water presented a higher prevalence of lung complications.<br><br>After allowance of water, a **significant** difference with a mean fluid intake between two groups (p<0.001).
Karagiannis M 2014	16	Pre-intervention (TF only)<br>Post-intervention (TF + free access to water + oral hygiene protocol (chorhexidine) prior to provision of water)<br>***TP not specified***	People with OD who are mobile and had relatively good cognitive ability were recruited.<br><br>Significant difference in fluid intake during pre and post intervention stages (p<0.001).<br><br>Remarkable differences for responses to questions related to hydration (p<0.001) and oral mouth care.<br><br>Access to water in addition to the prescribed modified diet results in a significant increase in overall fluid intake, when compared to the intake during the thickened fluid only period.

Garon B<br>1997	20	Control = 10 only TP<br>Intervention= 10 TP and free access to water<br><br>***TP not specified***	90% of the control group reported displeasure with TP and ALL reported that they desired water or ice chips for thirst.<br><br>ALL subjects reported that TP were not thirst-quenching and were lacking in taste and enjoyment
Vivanti A<br>2009	25	Mean intakes of water in OD adult patients.<br><br>**Guar gum**	Daily fluid intake from food was greater than from beverages whether receiving diet alone or diet with enteral or parenteral fluid support.<br>Individuals receiving fluid from oral intake only (foods and thickened beverages) fell short of reaching minimum calculated fluid requirements.<br>Inadequacy of fluid intakes and offers insight on the most important fluid sources in an at risk population group.<br>Mean fluid intakes from food and beverages alone fell below the calculated minimum fluid requirements each day for every patient.
McGrail A<br>2015	39	Thin liquids (G1) = 21<br>Thickened liquids (G2) =18<br><br>**Starch**	G1 had significantly larger **fluid offered and intaked** than G2.
McCormick<br>S 2008	11	Pre-thickened = 11<br><br>Group A= pre-thickened product<br>Group B= Thickened liquids<br><br>**Starch**	8/10 patients had higher fluid intake when taken in the form of pre-thickened fluids as compared to powder-thickened fluids (p = 0.47).
McGrail A<br>2012	30	Thin liquids = 10<br>Thickened liquids = 10<br>Healthy = 10	Healthy patients drank significantly more fluids than the ones receiving thin liquids (p=0.004) and TL (p=0.001).<br><br>Also a significant difference in mean fluid intake between thin liquids and TL.<br><br>More access to beverages in patients receiving thin liquids than TL.
